# EANM dosimetry committee series on standard operational procedures: a unified methodology for ^99m^Tc-MAA pre- and ^90^Y peri-therapy dosimetry in liver radioembolization with ^90^Y microspheres

**DOI:** 10.1186/s40658-021-00394-3

**Published:** 2021-11-12

**Authors:** Carlo Chiesa, Katarina Sjogreen-Gleisner, Stephan Walrand, Lidia Strigari, Glenn Flux, Jonathan Gear, Caroline Stokke, Pablo Minguez Gabina, Peter Bernhardt, Mark Konijnenberg

**Affiliations:** 1 Nuclear Medicine Unit, Foundation IRCCS Istituto Nazionale Tumori, Milan, Italy; 2 Department of Medical Radiation Physics, Lund University, Lund, Sweden; 3 Nuclear Medicine, Molecular Imaging, Radiotherapy and Oncology Unit (MIRO), IECR, Université Catholique de Louvain, Brussels, Belgium; 4 Medical Physics Division, IRCCS Azienda Ospedaliero-Universitaria di Bologna, Bologna, Italy; 5 Joint Department of Physics, Royal Marsden Hospital & Institute of Cancer Research, Sutton, UK; 6 Department of Diagnostic Physics, Oslo University Hospital, Oslo, Norway; 7 Department of Medical Physics and Radiation Protection, Gurutzeta/Cruces University Hospital, Barakaldo, Spain; 8 Department of Radiation Physics, Institute of Clinical Science, Sahlgrenska Academy, University of Gothenburg, Gothenburg, Sweden; 9 Department of Medical Physics and Biomedical Engineering, Sahlgrenska University Hospital, Gothenburg, Sweden; 10 Department of Radiology and Nuclear Medicine, Erasmus MC, Rotterdam, The Netherlands

**Keywords:** Radioembolization dosimetry, ^99m^Tc-MAA, ^90^Y microspheres, Lung shunt, Liver dosimetry, Tumour dosimetry, ^90^Y PET, Prospective/retrospective dosimetry

## Abstract

The aim of this standard operational procedure is to standardize the methodology employed for the evaluation of pre- and post-treatment absorbed dose calculations in ^90^Y microsphere liver radioembolization. Basic assumptions include the permanent trapping of microspheres, the local energy deposition method for voxel dosimetry, and the patient–relative calibration method for activity quantification.The identity of ^99m^Tc albumin macro-aggregates (MAA) and ^90^Y microsphere biodistribution is also assumed. The large observed discrepancies in some patients between ^99m^Tc-MAA predictions and actual ^90^Y microsphere distributions for lesions is discussed. Absorbed dose predictions to whole non-tumoural liver are considered more reliable and the basic predictors of toxicity. Treatment planning based on mean absorbed dose delivered to the whole non-tumoural liver is advised, except in super-selective treatments.

Given the potential mismatch between MAA simulation and actual therapy, absorbed doses should be calculated both pre- and post-therapy. Distinct evaluation between target tumours and non-tumoural tissue, including lungs in cases of lung shunt, are vital for proper optimization of therapy. Dosimetry should be performed first according to a mean absorbed dose approach, with an optional, but important, voxel level evaluation. Fully corrected ^99m^Tc-MAA Single Photon Emission Computed Tomography (SPECT)/computed tomography (CT) and ^90^Y TOF PET/CT are regarded as optimal acquisition methodologies, but, for institutes where SPECT/CT is not available, non-attenuation corrected ^99m^Tc-MAA SPECT may be used. This offers better planning quality than non dosimetric methods such as Body Surface Area (BSA) or mono-compartmental dosimetry. Quantitative ^90^Y bremsstrahlung SPECT can be used if dedicated correction methods are available.

The proposed methodology is feasible with standard camera software and a spreadsheet. Available commercial or free software can help facilitate the process and improve calculation time.

**Supplementary Information:**

The online version contains supplementary material available at 10.1186/s40658-021-00394-3.

## Preamble

The European Association of Nuclear Medicine (EANM) is a professional non-profit medical association that facilitates communication worldwide among individuals pursuing clinical and research excellence in nuclear medicine. The EANM was founded in 1985.

These guidelines are intended to assist practitioners in providing appropriate nuclear medicine care for patients. They are not inflexible rules or requirements of practice and are not intended, nor should they be used to establish a legal standard of care.

The ultimate judgment regarding the propriety of any specific procedure or course of action must be made by medical professionals taking into account the unique circumstances of each case. Thus, there is no implication that an approach differing from the guidelines, standing alone, is below the standard of care. On the contrary, a conscientious practitioner may responsibly adopt a course of action different from that set out in the guidelines when, in the reasonable judgment of the practitioner, such course of action is indicated by the condition of the patient, limitations of available resources or advances in knowledge or technology subsequent to publication of the guidelines.

The practice of medicine involves not only the science but also the art of dealing with the prevention, diagnosis, alleviation and treatment of disease.

The variety and complexity of human conditions make it impossible to always reach the most appropriate diagnosis or to predict with certainty a particular response to treatment. Therefore, it should be recognised that adherence to these guidelines will not ensure an accurate diagnosis or a successful outcome. All that should be expected is that the practitioner will follow a reasonable course of action based on current knowledge, available resources and the needs of the patient to deliver effective and safe medical care. The sole purpose of these guidelines is to assist practitioners in achieving this objective.

## Foreword

### Aim of this guideline

In the loco-regional liver treatment with ^90^Y microspheres, there is increasing evidence of correlation between the absorbed doses delivered and biological effects in terms of local lesion response, treatment-related toxicity and overall survival [1–3]. However, data are often collected from a variety of pathologies (primary or metastatic lesions), using different imaging methodologies (pre- or post-therapy single photon computer emission tomography (SPECT) or ^90^Y-positrom emission tomography (PET)) and evaluated according to ill-defined endpoints related to toxicity and response. This makes it difficult to compare results. The evidence of absorbed dose–effect relationships is clearly reported in several retrospective studies [2]. Indications of improved outcome after patient-specific dosimetric treatment planning are available in a study on sequential cohorts [4]. Strong evidence was also demonstrated recently in a prospective randomized multicentric trial [5]. However, additional research and a systematic overview is still required to reach a consensus in a field characterized by applications to many different tumour types with different kinds of microspheres and with different dosimetric criteria and methods [2, 5–9].

The present document does not cover dose–effect relationships nor provide absorbed dose thresholds for toxicity and efficacy. These aspects are beyond the scope of this standard operational procedure. It rather focuses on the first step of the chain, the absorbed dose evaluation, to aid in the standardization of methodology, and proposes a common framework for dosimetric data collection.

In this document, recommended methods are supported providing a short review of the available literature for each topic. Where no published methodology nor data were available, recommendations are made based on internal consensus and the knowledge of some Committee members experienced in the field (“internal consensus”).

The American Association of Physicists in Medicine (AAPM) published multidisciplinary recommendations in 2011 [10]. Additional information about physical aspects in radioembolization may be found in recent review papers [11–13]. Clinical guidelines for liver microsphere treatment were produced in 2011 by the EANM Therapy Committee [14]. The American College of Radiology (ACR) revised clinical guidelines in 2019 (https://www.acr.org/-/media/ACR/Files/Practice-Parameters/rmbd.pdf).

### Basic assumptions and definitions for microsphere dosimetry

A fundamental assumption for microsphere dosimetry is the permanent trapping of these medical devices (i.e. the absence of biological clearance). This assumption has been scarcely investigated. Post-transplantation and post-mortem microscopy of liver sections showed preferential uptake of microspheres (2 glass and 2 resin patients) surrounding the tumours, in the periphery of the triad units within medium to small calibre arteries, confirming other clinical and preclinical work [15–17]. Consequently, unlike radiopharmaceuticals administered systemically, there is unanimous consensus throughout the literature that it is sufficient to acquire only a single imaging time point for microsphere dosimetry, with the time activity curve completely governed by the physical decay.

Images acquired both for simulation and verification are generally a standard aspect of the treatment protocol. Therefore, any additional resource required for dosimetry is limited to the image processing and calculation. Dedicated dosimetric software are also not strictly required.

The simplified segmentation model behind microsphere dosimetry assumes that liver can be divided in distinct volumes of interest (VOIs) corresponding to different sets of cells, or compartments (See the Segmentation section).

The first two compartments are non-tumoural liver tissue and lesions. The former includes the whole functional liver tissue, and excludes non-functional regions from previous treatments or cysts. Non-tumoural whole liver can be subdivided into the region perfused by the radioactive particles (target non-tumoural liver), and that non perfused by radioactive particles (non-target non-tumoural liver).

Lesions can be grouped in target and non-target lesions, depending on their location within or outside of the injected segments. In each lesion, three further regions may be defined. The CT lesion (i.e. the morphological region defined on CT (usually on the arterial phase of a contrast-enhanced CT)) can also be split into a high-perfusion region and low-perfusion region. The level of perfusion may pertain to the concentration of contrast medium on radiological images or to that of the radioactive particles in the nuclear medicine images. Since the absorbed dose is delivered by radioactive particles, in the rest of this document, “perfusion” is meant as “perfusion by radioactive particles”.

The final VOI is the tumoural portal vein thrombus, which has to be dosimetrically evaluated. This may be partially or completely external to the liver.

This segmentation model is simplified, and more complex situations may arise where the attribution to tumour versus non-tumour compartment is difficult, and where the model fails. Examples include infiltrative lesions, with a mixture of healthy hepatocytes and tumoural cells (see the Image segmentation of infiltrative lesions section), or large centro-hepatic lesions belonging to both right and left lobes and fed by two arteries. In such a case, a lobar administration targets only a portion of this lesion..

## Three kinds of “microspheres”

### Physical properties of particles in ^90^Y radioembolization

Table 1 reports the physical properties of the particles used in radioembolization planning and treatment with ^90^Y microspheres, indicating parameters of interest within a dosimetric context.

**Table 1:** Physical characteristics of MAA and ^90^Y microspheres

	^99m^Tc-MAA^Δ^	Resin spheres^♣^	Glass spheres^♠^
**Commercial name**		SIR-Spheres®	TheraSphere®
**Manufacturer**		Sirtex Medical	Boston Scientific
**Diameter (μm) ± standard deviation (μ﻿m)**	31.2; Range (10–100) non spherical	32.5 ± 2.5; range (20–60)	25 ± 5
**Specific gravity**	n/a	1.6 g/mL	3.6 g/mL
**Vial activity (GBq)**	1.5 (α)–3.7 (β)	2.5–10**	From 3* to 20*, with 0.5 step
**Activity per particle (Bq)**	333 (α)–822 (β)	42–166**	4354*, 1539^♦^, 544^♥^
**Millions of particle per GBq**	3 (α)–1.2 (β)	24–6**	0.23*, 0.65^♦^, 1.84^♥^
**Millions of particles in a typical administration**	0.3–0.7	1.2 GBq 28.8–7.2** millions	2.6 GBq 1.7^♦^ 4.8^♥^ millions
**Stasis phenomenon (backflow, jammed injection)**	Never reported	Reported	Never reported
**Material**	Human albumin	Resin with ^90^Y on surface	^90^Y in glass matrix
**Shelf life**	6 h from labelling	(− 4, + 1) days	12 days
**Handling vial for dispensing**	Required	Required	Not required
**Vial fractionation in syringe**	Required	Required	Not required
**Iodinated contrast medium during administration**	Not required	Recommended	Not required

(α) 4.5 millions of MAA labelled with 1.5 GBq

(β) 4.5 millions of MAA labelled with 3.7 GBq

* Measured, at the reference date. [18]

**Depending on the injection time. Resin microspheres can be delivered up to 4 days before the calibration date, when vial activity is 10 GBq. This means that the planned activity can be injected with one third of the number of particles with respect to the calibration date (https://www.sirtex.com/eu/clinicians/flexdose-delivery-programme/)

^♦^Four days after the reference time.

^♥^Eight days after the reference time.

^Δ^www.nuclearonline.org/PI/mallinckrodt%20MAA.pdf, www.accessdata.fda.gov/drugsatfda_docs/label/2009/017881s010lbl.pdf

^♣^https://www.sirtex.com/media/169513/pi-ec-14-spheres-ifu-eu-row.pdf

^♠^https://www.bostonscientific.com/en-US/products/cancer-therapies/therasphere-y90-glass-microspheres.html

Albumin macroaggregate properties are also reported [19].

^90^Y is a β^-^ emitter with a physical half-life T_1/2_^phys^ of 64.042 ± 0.031 h. Maximum and mean β^-^ energy are E_β_^MAX^(^90^Y) = 2280 keV and E_β_^MEAN^ (^90^Y) = 933.7 keV. (http://www.nucleide.org/DDEP_WG/Nuclides/Y-90_tables.pdf). These correspond to a maximum range in tissues of 11 mm with 90% of energy deposited within 4.9 mm.

Recently, ^166^Ho microspheres became commercially available, and the respective dosimetry is yet to be extensively studied [20]. For this reason, dosimetric methods regarding ^166^Ho microspheres are outside the scope of this document; in addition, dosimetry of other experimental microspheres are not considered.

### Leaching of ^90^Y activity

The fraction of administered activity dissociating from microspheres and measured in urine after 12 h is reported as 0.066% for resin and 0.0025% for glass microspheres respectively [21]. A more recent paper obtained the maximal percentual excretion in 48 h equal to 0.1% (resin), 0.01% (glass) and 0.005% (^166^Ho) [22]. According to ICRP30 and ICRP134 [23], approximately 25% of free Yttrium will be excreted through the urine and 40% taken up in bone. A leaching factor ten times higher than these values into blood would be insufficient to cause any deterministic or stochastic effect to the red marrow.

## Pre-therapy simulation

### Prediction of ^90^Y microsphere distribution using ^99m^Tc-MAA

The potential to perform accurate dosimetric treatment planning relies on the predictive accuracy of ^99m^Tc-MAA for ^90^Y microsphere distributions. The difference in size, shape and number between MAA and therapeutic microspheres is considerable (Table 1).

For resin microspheres, several studies demonstrated difference between ^99m^Tc-MAA distributions and microsphere therapeutic distributions imaged using ^90^Y bremsstrahlung SPECT [24–26]. However, the absence of scatter correction in ^90^Y imaging put doubt on the accuracy of the correlation metrics. Kao et al., with ^90^Y PET, supported the notion that the MAA distribution can be predictive. However, a maximum deviation in tumour-absorbed dose of ~ 20% and average of ~ 6% was reported [27]. A potential source of poor correlation between MAA and resin microspheres in these works was stasis or backflow, generated during the treatment administration. A marked decrease in the stasis rate can be achieved using 5% glucose solution as demonstrated by Ahmadzadehfar et al., who obtained a reduction from 28 to 11% of stasis cases [28]. Despite this expedient, the difference between MAA prediction and therapy can still be considerable, especially for lesions, both primary and metastatic, as reported by Jadoul et al. [29] and by Richetta et al. [30].

For glass spheres, large dosimetric discrepancies were also reported [31–34], and more severe when a larger number of microspheres per GBq were injected (due to a longer decay interval from calibration date) [35].

In addition to the importance of catheter tip repositioning, studies with ^166^Ho microspheres helped to understand that a difference in size and shape between MAA and therapeutic microspheres play a major role in prediction accuracy [36, 37]. This observation can be applied to ^90^Y microsphere. The factors that affect the mismatch between the simulation with MAA and the therapy distribution are to some extent intrinsic and to some extent operator-dependent (explicitly marked with (*)):

- Uncertainty about the stability of MAA after labelling (*)
- Different catheter positioning (*), both longitudinally and radially, especially in proximity to an arterial bifurcation, see [25–27]
- The kind of catheter (*) [38]
- The speed of injection (*)
- The induction of vessel spasm due to a high number of therapeutic particles or a prolonged angiographic procedure (*) [39]. Furthermore, fragile vessels may be damaged in the diagnostic procedure which is generally more prolonged than the treatment session
- The induction of vessel spasm by the flushing medium (sterile water) (*), see [28]
- A prolonged time interval between simulation and therapy (*) that could allow tumour progression
- Different size distribution and shape of injected particles between the ^99m^Tc-MAA simulation and the therapy session [27, 36]
- Different number of injected particles between the ^99m^Tc-MAA simulation and the therapy session [40, 41], i.e. different volume of injected particles (this depends markedly on the kind of microspheres)
- Different specific gravity of the MAA and the therapeutic particles, see Table 1
- Different tumour types with different degree of vascularisation (HepatoCellular Carcinoma (HCC) versus metastatic disease)
- Size of tumour**:** higher risk of reflux in small lesions, partial volume effect in lesions < 2 cm

### Importance of prediction with ^99m^Tc-MAA

A user should try to minimize dosimetric discrepancies between prediction and therapy with careful control of the operator-dependent factors described above. Until a better predictor becomes available, the use of ^99m^Tc-MAA for therapy optimization is however encouraged for two reasons:

1. Correlations between the absorbed dose and average clinical outcome of a population (toxicity rate, response rate, progression-free survival (PFS), overall survival (OS)) were established in studies based on ^99m^Tc-MAA dosimetry [1, 2, 4, 5]. This can be explained as, according to all authors, the mean difference (the bias in the Bland -Altman plot) is negligible.
2. ^99m^Tc-MAA dosimetry provides a better treatment planning method compared with activity prescriptions based on the Body surface area (BSA) method, or based on mono-compartment dosimetry methods [42].

MAA dosimetry has been demonstrated to improve the average quality of treatment. However, it is the opinion of the Committee that the decision to treat an individual solely on the bases of the predicted lesion-absorbed dose is a delicate matter [43].

According to all authors, reported dosimetric differences between MAA prediction and verification are significantly larger for lesions than for normal tissue. This is true for both kinds of microspheres. Therefore, the more reliable prediction to non-tumoural tissue should be the primary planning criterion, without neglecting lesion-absorbed dose prediction [6, 31, 33, 34, 42]. See the Treatment planning section using mainly non-tumoural whole liver dosimetry for additional information about this argument.

### ^99m^Tc-MAA image acquisition and timing

EANM clinical guidelines recommend ^99m^Tc-MAA examinations prior to any ^90^Y microsphere treatment to investigate the possible presence of shunting [14]. The following workflow is recommended. All patients are first examined by planar imaging to evaluate the possible lung shunt fraction (LSF_planar_). SPECT/CT covering the liver/abdomen should be performed for all patients, as this is required to exclude from treatment patient showing any gastrointestinal shunt [14]. This scan can be conveniently used for the purpose of dosimetry-based treatment planning. For patients where a substantial lung shunt is present, the SPECT/CT field of view should cover both the liver and whole lungs, with a second SPECT scan if necessary, to accurately quantify the LSF_TOMO_. Dittman et al. [44] provided a cut-off of LSF_planar_ > 10%. The clinical importance of this evaluation has been emphasized by others, see review by Gill & Hiller [45], For patients where the LSF_planar_ is < 10%, the additional lung SPECT/CT can be omitted. See How to handle the size and number of lesions section and The proposed procedure: three classes of lung shunt section for details.

#### Planar scintigraphy for lung shunt determination

A static planar image (200 s or less) or fast whole-body scan (typically 12 cm/min or more) should be acquired, preferably utilising the conjugate view technique, within 1 h or less after ^99m^Tc-MAA administration. The images should include both thyroid and urinary bladder as the visibility of these organs is an indication of ^99m^Tc detachment from MAA. Free ^99m^Tc also appears as stomach uptake which can complicate the determination of any gastric shunt. Perchlorate administration prior to the MAA scan helps prevent gastric uptake of free ^99m^Tc [14], aiding in the interpretation of any potential gastric shunt. Scintigraphy should be acquired with a gamma camera equipped with low-energy collimators, matrix size 256 × 256 or 128 × 128, and an energy window centred at 140 keV.

#### SPECT/CT acquisition

Dosimetric SPECT acquisitions should be corrected for attenuation using a hybrid SPECT/CT system. In absence of such a system, a CT attenuation corrected SPECT image can be generated by co-registration of the SPECT with the diagnostic CT scan acquired at similar time to the SPECT scan. Care should be taken to accurately reproduce the patient position between scans. For both applications, the effect of density override by the contrast media should be corrected. See Image co-registration section for co-registration options.

Breathing motion can cause a significant issue for segmentation, since a mismatch between CT and SPECT can occur at the dome of the liver. Several solutions are proposed [11], the simplest of which is presented in the Image segmentation section.

In addition to CT-based attenuation correction, the reconstruction of SPECT images should include scatter correction, preferably within reconstruction loop. Lack of scatter correction can result in an overestimation of up to 40% in the absorbed dose to non-tumoural tissue [42]. The choice of scatter correction method is less critical, with adequate results demonstrated across all common approaches [46]. Resolution recovery is also advised to reduce the partial volume effect (PVE) in small lesions. A typical SPECT acquisition protocol is summarised below:

- A range of 75–150 MBq of ^99m^Tc-MAA is administered in the proper hepatic artery [14]
- Patient with raised arms (but according to patient’s compliance). If a patient is unable to keep his arms raised for the exam duration, the usual voltage (110–120 kV) should be increased to the maximal available (130–140 kV) to mitigate the CT artefacts caused by the alignment of humera and spine
- LEHR or LEUHR collimators.
- Angular sampling of 3°
- Image matrix of at least 128 × 128
- Emission energy window centred at 140 keV, with a width of 15%
- Scatter energy window adjacent at left side of the emission energy window, with a width of one half of the emission energy window in case of manual subtraction of projections
- Non-circular orbit, automatic body contouring
- At least 15 s per angular step, preferably 20 s or longer, to reduce noise for voxel dosimetry. An experimental methodology decreasing the total scan time to 10 min was proposed [47].

Use of additional contrast medium for the SPECT/CT acquisition can be helpful, but could be clinically contraindicated if acquired at a short time interval after the diagnostic CT imaging session. In the case of dual axial field of view (AFOV) SPECT/CT to cover lungs, the time per projection, as well as all other acquisition parameters, should be identical in the two scans.

### Optimization of SPECT image reconstruction

Reconstruction protocols for quantitative SPECT may differ from that of diagnostic examinations and should include corrections of physically degrading effects such as attenuation, scatter, and collimator-response compensation. The impact of these effects was analysed by means of Monte Carlo simulations by Pacilio et al. [46], who found the most pronounced degrading effect to be the PVE and respiratory motion, leading to underestimation of lesion-absorbed doses. With 7 mm FWHM ^99m^Tc SPECT/CT spatial resolution, the loss in activity due to PVE was experimentally determined to be > 20% for spheres of 1.8 cm in diameter [48]. In voxel dosimetry, lesions with a diameter < 2 cm should be excluded from analysis since PVE cannot be corrected in this application [48].

Noise can significantly impact the accuracy of voxel dosimetry, as shown by Cheng et al. [49]. Optimisation of the reconstruction is therefore needed both in mean dose evaluation and in voxel dosimetry, to obtain the best compromise between low PVE (high recovery coefficients for small objects) and noise. The number of required iterations and subsets are generally higher than that used for diagnostic purposes [48, 49]. The potentially higher noise level resulting from this choice is mitigated by the high counting statistics of the high activity concentration from loco-regional injections (^99m^Tc 150 MBq/1 L).

Pre- or post-filtering should be avoided since it reduces spatial resolution and increases PVE.

Optimal ^99m^Tc SPECT/CT reconstruction parameters should be preliminarily determined on each specific system using two phantom acquisitions (a Jaszczak phantom with uniform concentration and another with hot spheres are is recommended), with the aim to measure noise and recovery coefficients as a function of the number of updates P (number of iterations multiplied by the number of subsets). As an example, see MIRD pamphlet No. 23 [50]. The procedure for reconstruction optimization is described in the ^99m^Tc Phantom scans for reconstruction optimization section in the Appendix and in [48, 51].

#### Missing CT-based attenuation correction

In centres where CT-based attenuation correction is not possible, non-attenuation corrected SPECT images can be used for calculation of the mean absorbed dose in non-tumoural whole liver and lesions, not in lung. This is possible since activity quantification by the patient–relative conversion factor (Image quantification section) partly corrects for attenuation [42, 46].

## Post-therapeutic verification

Post-therapeutic imaging is clinically useful to determine any mismatch between simulation and therapy sessions (reported in the Prediction of ^90^Y microsphere distribution using ^99m^Tc-MAA section).

Potential considerations are cases of inadvertent and potentially toxic distributions (i.e. gastroduodenal uptake), which can be clinically handled with immediate pharmacological therapy, endoscopic procedures or delayed surgery. In addition, cases of unsatisfactory ^90^Y distributions can potentially be corrected using a three-step procedure proposed by Bourgeois et al. [52].

From a dosimetric perspective, the actual delivered absorbed doses are of interest to improve the understanding of absorbed dose–effect relationships.

Quantitative ^90^Y bremsstrahlung SPECT is challenging and requires dedicated and detailed correction methods (see Dosimetry based on ^90^Y bremsstrahlung SPECT section in the Appendix). The superior quantitative accuracy of ^90^Y PET/CT was first indicated by Lhommel et al. [53]. According to Elschot et al. [54], PET outperforms bremsstrahlung SPECT/CT. On a phantom with spheres, they demonstrated a dose underestimation ranging from 45% (10-mm sphere) to 11% (37-mm sphere) with ^90^Y TOF PET/CT, versus 75–58% with ^90^Y bremsstrahlung SPECT/CT, though noise level in PET images is markedly higher. Superior PET contrast recovery coefficients were confirmed after a PET reconstruction giving the same noise level as bremsstrahlung SPECT. Takahashi et al. [55] used Monte Carlo simulated data to calculate the contrast recovery coefficient and, more importantly, the contrast to noise ratio as index of lesion detectability. Spheres of the NEMA phantom with background ratios of 40:1, 20:1 and 10:1 were considered. Superior contrast recovery coefficients were obtained for ^90^Y PET. However, the PET noise became excessive for a background concentration < 100 kBq/mL, corresponding to an absorbed dose of 5 Gy for the 10:1 concentration ratio, thus giving a poorer contrast to noise ratio. Superior ^90^Y PET spatial resolution is remarked in a case report by Kao et al. [56].

Therefore, despite some imaging limitations, ^90^Y PET/CT should be the dosimetry imaging modality, if physically available, in centres lacking special correction methods for ^90^Y bremsstrahlung SPECT. In the Appendix you find details about dosimetry based on ^90^Y bremsstrahlung SPECT.

### Measurement of the ^90^Y injected activity

Careful measurement of the administered ^90^Y activity is important to comply with the therapy prescription and to provide the basis for an accurate dosimetry calculation. The topic of ^90^Y microsphere activity measurements is specifically covered by the AAPM guideline [10]. Uncertainty regarding administered activity should be considered to evaluate the global dosimetric uncertainty budget [57].

The activity within the shipping vial of resin microspheres is certified to be within ± 10% [10], and the first three shipped vials are used for the calibration of the activity meter. The activity transferred to the V-vial is then determined by difference in the activity meter measurements of the shipping vial before and after the transfer. The relative exposure rate from the V-vial before and after the injection indicates the residual activity, with the detector placed in contact on the surface of the administration box. Initial and final exposure rate are obtained as geometric means of reading on opposite sides of the administration box. Contamination of the administration box itself is included in these measurements as residual activity.

Ideally, residual activity within the catheter should also be taken into account. The authors have observed up to and an extra 90% residual activity within the catheter. Based on these experiences, it is recommended that the exposure rate measurements be made with the catheter placed inside the administration box in addition to the V-vial.

For glass spheres, the manufacturer calibrator measurements are routinely verified with NIST, for the full range of dose sizes [10]. It is therefore recommended to use this activity value at reference time for any proceeding calculations. As indicated in the user manual, the residual activity is determined as the ratio of the exposure rate at a fixed distance from the shielded plastic vial and from the PMMA cylindrical waste box, containing any waste accumulated during administration. Rates are measured before and after the administration. The vendor provides a sheet of paper where the shielded vial and waste box are positioned at a reproducible fixed distance from a portable dose rate monitor. It is suggested that radioactive and potentially infected waste be placed in the single-use bin which can fit inside the provided PMMA waste box, to avoid radioactive and biological contamination of the latter (internal consensus).

For both kinds of spheres, the residual non-injected activity is important especially in cases of inadvertent partial administration for stasis or after an incident with the injection device. In the following, the injected activity refers to the net injected activity (i.e. the shipped activity subtracted by residual activity).

### Timing of post-therapy verification

Given the low abundance of photons, both ^90^Y bremsstrahlung SPECT and ^90^Y PET would benefit from immediate imaging after the therapeutic administration, to avoid a decrease in count rate due to physical decay, which is about 30% per day. However, priority should be given to patient safety. After the femoral artery puncture, it may be safer to wait until the following day when the patient is able to walk freely. Longer delays should be avoided. In case of radial puncture, this precaution is not necessary, and the ^90^Y scan can be acquired in the same day of the administration.

### ^90^Y PET/CT acquisition

Although the probability of a positron emission from ^90^Y is very low (3.186 ± 0.047) × 10^-5^ [58], dosimetry using ^90^Y PET/CT imaging is feasible in clinical routine [59–64]. For hospitals with multiple PET/CT scanners, the system with the highest sensitivity should be used. The sensitivity of 2D PET is insufficient.

On older scanners, ^90^Y may not be present within the list of common radionuclides. In such a case, an acquisition using the pre-set from an alternative long-lived PET emitter can be used, such as ^22^Na, or ^68^Ge. This is required to avoid overcorrection of isotope decay between bed positions and between activity measurement time and scan acquisition time. When using a ^22^Na pre-set, absolute quantification can be obtained by entering into the acquisition software the injected ^90^Y activity multiplied by 3.186 × 10^-5^. The patient relative calibration method bypasses this issue (see the Image quantification section).

The duration of the PET scan should be as long as reasonably possible. A copper ring within the gantry to reduce the amount of random coincidences from bremsstrahlung photons was initially proposed, but is now considered contra indicatory [65]. For a typical 3D PET scanner, with a 15-cm AFOV, a minimum15-min per-bed position should be acquired [59]. To compensate for the sensitivity loss at the edges of the AFOV, the overlap between adjacent bed positions should be increased above that commonly used for ^18^F imaging, typically to 15 or 17 slices. In the absence of substantial lung shunt, two bed positions are usually required to cover the whole liver. In the presence of substantial lung shunt, the AFOV should cover both liver and lungs which may require three bed positions. An image matrix size of 128 × 128 is sufficient.

### ^90^Y PET reconstruction

The QUEST international multi-centre study provided important indications regarding ^90^Y imaging [66]. Sixty-nine non-digital PET scanners were used to acquire images several times during the decay of a ^90^Y-filled NEMA 2007/IEC 2008 PET body phantom. The main results from the study were as follow:

- The activity of the largest sphere was consistently underestimated by 10 to 20%, thus indicating the general suboptimal quantification accuracy of the non-digital technology of ^90^Y PET. These results were obtained using absolute system calibration.
- Systems equipped with TOF capability and resolution recovery (RR) yielded better results than their counterparts without these features.
- On one scanner brand, the total activity in the phantom could not be quantified within the ±10% tolerance, from 1.5 down to 0.5 GBq. However, the activity concentrations investigated (0.15–0.05 GBq /L) were considerably lower than those encountered in clinical practice, typically around 1.2–2.6 GBq /L.
- Lesions smaller than approximately 2 cm in diameter (i.e. < 4.2 cm^3^ in volume) exhibit pronounced PVE.

In principle, PVE corrections using recovery coefficients can be applied for mean dose evaluation. However, caution is still required when reporting doses to lesions smaller than 2 cm in diameter, due to large uncertainties and inaccuracy in volume determination and consequent PVE correction (internal consensus).

When performing voxel dosimetry (Voxel dosimetry section), noise reduction is important. In ^90^Y PET, the main contribution to noise is that due to low image statistics. Unlike ^99m^Tc SPECT, optimization of the ^90^Y PET reconstruction protocol will not adequately reduce noise [67, 68]. Reconstruction with TOF, resolution recovery and without additional filtering is preferable. This will generally result in a noisier image. However, the activity recovery is higher for small spheres [69]. Due to the emission of bremsstrahlung photons, the trues/randoms ratio is considerably lower than ^18^F-FDG imaging, and corrections for randoms are more critical. Delayed random sinogram subtraction is not recommended since it may lead to an under-correction, and thus overestimation of activity [66, 70]. The ^90^Y PET reconstruction protocols section in the Appendix gives practical advice about reconstruction settings for some non-digital PET scanners.

## Image co-registration

### Co-registration for proper segmentation

VOI delineation (i.e. segmentation of images) is a critical requirement for dosimetry. Considerable uncertainties can be introduced into the estimated absorbed doses, due to the image characteristics, the physical aspects that underlie the tomographic image formation, and operator dependencies in the contouring. The delineated VOIs should always be approved by the physician responsible for the treatment.

**Fig. 1:**
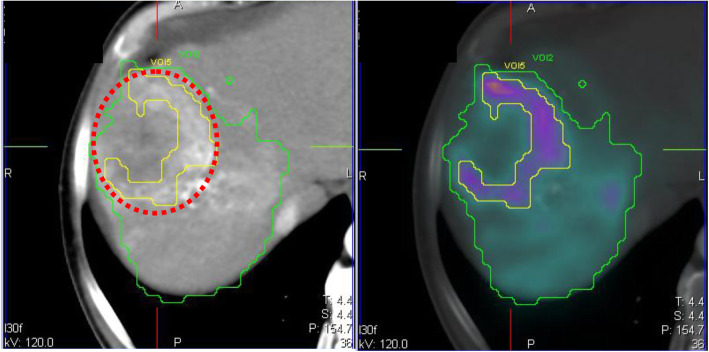
Segmentation of a lesion with non-perfused core, based on ^99m^Tc-MAA SPECT image (in colour on the right) co-registered to a contrast-enhanced CT (in black and white in both panels) (manual rigid co-registration). The lesion is segmented both on CT (red dotted line) and nuclear medicine (yellow line) images. The region “liver perfused” with radioactive particles is segmented on nuclear medicine images (green line). For toxicity prediction, the CT-based segmentation (red dotted) should be considered as lesion has to be excluded from the non-tumoural whole liver volume

Due to the complexity of the activity distribution often seen in liver disease, a reliable segmentation requires the use of co-registered radiological and nuclear medicine images displayed as fused images. Without fused images, it is otherwise difficult to precisely delineate lesion borders, due to mismatch between morphological and perfused regions (Fig. 1). Image quality of hybrid systems (SPECT/CT and PET/CT) is often inadequate since the CT is not contrast-enhanced. Diagnostic contrast-enhanced radiological images help, although segmentation may still be difficult on infiltrative hepatocellular carcinoma lesions or in cases of poor vascularisation of metastases. In the latter, contrast-enhanced MRI or ^18^F-FDG PET images can be used.

### Images for co-registration

Absorbed dose calculations rely on the count distribution in the nuclear medicine image. It is therefore imperative that voxel values are not altered during the co-registration process. For this reason, nuclear medicine images should not be re-interpolated (i.e. translated, rotated), but above all not deformed, especially in voxel dosimetry. Rather, it is recommended that the anatomic image should be moved or deformed.

Registered images should always be visually inspected. For hybrid SPECT/CT and PET/CT systems, the simplest approach is to use the available co-registered CT image to aid image segmentation.

While automatic registration methods, such as those based on the mutual information metric, often fail to register images with very different characteristics, a manual rigid CT-to-SPECT registration is acceptable for attenuation correction purposes, since the attenuation map has a low level of detail. However, for the delineation of small lesions at the liver–lung interface, deformable registration may be required.

To exploit the informative content offered by radiological imaging, a recently acquired contrast enhanced diagnostic CT scan (or MRI) can be registered to the CT acquired during hybrid imaging. Registration may be rigid, or including more detailed spatial transformations, and can often be performed automatically.

Dosimetric verification of the ^90^Y microsphere distribution is simplified by co-registration of the ^90^Y PET image to the attenuation and scatter corrected ^99m^Tc-MAA SPECT image. Usually, the patient position is similar in the two acquisitions, and the image characteristics are similar, making an automated rigid registration possible. Unavoidable PET voxel interpolation will likely occur, but without significant deformation. Comparison of the ^99m^Tc-MAA and ^90^Y microsphere distributions is then simplified since VOIs drawn on the SPECT image can be copied to the PET image. This method avoids a second segmentation, thus reducing the workload and additional uncertainty.

If the treatment indication is metastatic disease, an ^18^F-FDG PET/CT is useful to define the metabolically active portion of lesions. Co-registration of ^18^F-FDG PET-CT to ^99m^Tc-MAA-SPECT/CT can be achieved using the respective CT datasets [71].

Jafargholi Rangraz et al. [72] developed a semi-automated co-registration method using ^99m^Tc-MAA SPECT/CT, ^18^F-FDG PET/CT, and cone beam CT images to obtain automated segmentation of the liver, injected lobe, and lesions.

### Problems related to organ displacement and breathing **motion**

Organ displacement between different imaging sessions and breathing motion during hybrid imaging can introduce difficulties during co-registration.

The region that forms the basis for co-registration may be limited to a volume that includes the perfused portion only, while neglecting residual misalignment of non-perfused areas (local co-registration).

**Fig. 2:**
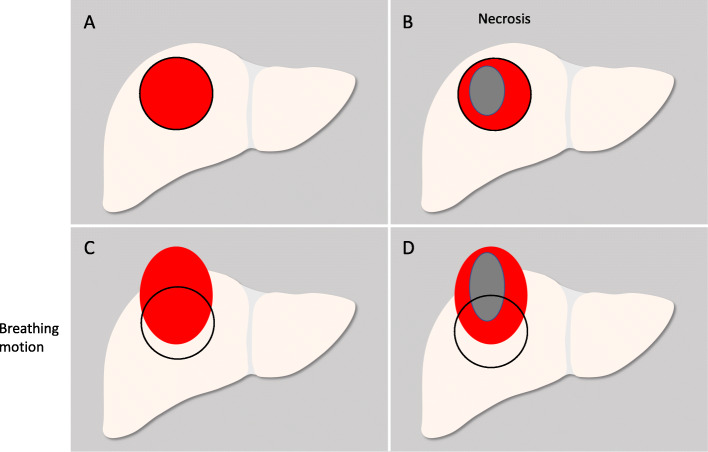
Schematic coronal slice of the two major difficulties that are encountered for segmentation: breathing motion (**C** and **D**) and tumours with low-perfusion regions (**B** and **D**)

Breathing motion often introduces large mismatch at the liver dome (Fig. 2). This motion induces spill-out of counts in the nuclear medicine image. Hence, it is often difficult to objectively delineate a VOI and determine the respective counts. The apparent LSF may also be exaggerated. In addition, this motion blur can cause an underestimate of absorbed dose to small lesions, where volumes may appear larger in the nuclear-medicine scan. This is further detailed in the next section.

For ^99m^Tc SPECT, studies of the respiratory motion under fluoroscopy are under development, but are not currently commercially available [73, 74]. For ^90^Y PET, gating is challenging, given the low abundance of annihilation photons, although a case study has reported that this might be feasible [75]. A possible improvement could be obtained using a gated CT, if the performance of the CT scanner is adequate [76]. Possible solutions are presented in the next section.

## Image segmentation

Segmentation is often a time-consuming but unavoidable requirement for treatment planning, as in external beam radiotherapy (EBRT). Although semi-automatic segmentation methods are being developed, the following instructions are intended for centres with standard nuclear medicine processing software and without dedicated dosimetry software.

### The two VOI method

Image choice will influence the accuracy of segmentation (Figs. 1 and 2). CT, particularly if contrast-enhanced, allows for accurate volume determination (“CT-based segmentation”), while nuclear medicine images (“NM-based segmentation”) allow for the identification of the perfused region of lesions, where a better correlation between absorbed dose and response is observed.

The use of both registered imaging modalities is advised, justified by the results of Garin et al., using a SPECT-based, CT-adapted segmentation of the injected region [77].

The drawback of lesion segmentation based only on a nuclear medicine image is that potential low-perfusion regions may be attributed to functional liver. A second CT-based segmentation is therefore necessary to confirm region allocation.

In conclusion, segmentation in the presence of large, partially perfused lesions, as in Fig. 2, should be defined both with CT-based and NM-based segmentation (internal consensus), i.e. evaluated in two VOIs for each lesion:

- A CT-based contour to be subtracted from total liver
- A threshold or manual contour based on SPECT for the perfused region of lesions

A similar two-VOI approach is suggested for object mismatched by breathing motion.

#### Image segmentation of infiltrative lesions

Infiltrative lesions present a difficulty, as they constitute a mixture of tumour cells and healthy hepatocytes which are both irradiated due to the crossfire effect of ^90^Y. Two potential approaches are proposed.

The simplest and safest approach is to consider the whole liver (including infiltrative lesions) as non-tumoural liver (i.e. not to define the infiltrative lesion borders). The absorbed dose to the non-tumoural whole liver will likely be overestimated, resulting in a reduction in prescribed activity. This is a safer treatment regimen. This agrees with the observation that infiltrative tumours, often accompanied by portal vein occlusion, are risk factors for toxicity [78].

Depending on the clinical situation of the patient (tumour involvement, degree of cirrhosis and liver function), a more aggressive approach may be attempted. Lesion segmentation can be performed using a threshold on SPECT images, without correspondence to CT. This approach requires careful consideration, since it introduces a new experimental method of defining the tumour extent based on MAA perfusion. Inevitably, this approach will predict lower non-tumoural liver-absorbed doses than the former, thus leading to higher administered activities. This method is currently under evaluation. Kokabi et al. [79] proposed contrast-enhanced MRI to define infiltrative lesion border.

### Procedure for image segmentation with three liver compartments

#### How to handle the size and number of lesions

Dosimetry requires segmentation of several compartments: lesion(s), their perfused portion, non-tumoural whole and perfused liver and lungs. Any non-functional liver volume should be subtracted from the liver to obtain the non-tumoural whole liver volume. In the case of multiple lesions, this process is cumbersome and time-consuming. Notably, there are often target and non-target lesions, depending on whether they are located within the injected liver portion or not. Non-targeted lesions are not evaluated with dosimetry, but their volume should be subtracted. We suggest counting the number of non-targeted small-volume lesions (< 4.2 mL or diameter < 2 cm) and to estimate total volume relative to the whole liver volume. An approximation of the non-tumoural liver volume will avoid lengthy volume outlining and/or dosimetric evaluations of many small lesions.

#### Segmentation sequence

Figure 2 illustrates the two principal problems that may be encountered during segmentation: displacement due to breathing motion and a lesion with a region of low perfusion. The Committee agreed upon the segmentation sequence detailed below. The complete segmentation sequence is only necessary if there is a mismatch between the CT and the border observed on the nuclear medicine image (Table 2). In cases of good overlapping between radiological and nuclear medicine contours (Fig. 2A), the sequence can be simplified.

**Table 2:** Regions to be segmented in complex situations of marked mismatch between radiological and nuclear medicine images, like Figs. 2B, C and D. Use a contrast-enhanced CT co-registered with CT of SPECT if necessary (see also Numerical example section in the Appendix)

Region number	Tissue region	VOI name	Segmentation aim and strategy
1	Whole liver	VOI_CT_(whole liver)	CT-based, to quantify volume. Exclude cysts or necrosis from previous treatments.
2	Targeted lesions	VOI_CT_(#1) VOI_CT_(#2) VOI_CT_(#3)	CT-based, including low-perfusion region to quantify volume. Derive respective counts from perfused lesion regions.
3	Non targeted lesion	VOI_CT_ (non-targeted lesions)	CT-based, including low-perfusion region
4	Perfused liver	VOI_SPECT_(PL)	SPECT-based, to determine counts
5	Perfused liver	VOI_CT_(PL)	To determine PL volume. To obtain this VOI, Crop 4 within 1
6	Perfused lesion regions	VOI_SPECT_(#1) VOI_SPECT_(#2) VOI_SPECT_(#3)	SPECT-based (aided by CT), excluding low-perfusion region , to determine SPECT lesion volume and counts
7	Perfused non-tumoural liver	VOI_SPECT_(PNTL)	To obtain the number of counts N. 4 minus 6: *N*{*VOI*_*SPECT*_(*PL*)} − *N*{∑_*i*_*VOI*_*SPECT*_(#*i*)}
8	Perfused non-tumoural liver	VOI_CT_(PNTL)	To obtain volume. 5 minus 2: VOI_SPECT_(PL) − ΣVOI_CT_(#i), limited within 1
9	Non-tumoural whole liver (includes also non-perfused liver)	VOI_CT_(NTL)	Non-tumoural whole liver volume is obtained by subtraction: Volume 1 minus (Volume 2 + Volume 3) Non-tumoural whole liver counts 4 subtracted of counts in 6 identical to 7 Record the non-tumoural liver-treated volume fraction V_f_ ${\boldsymbol{V}}_{\boldsymbol{f}}=\frac{{\mathbf{VOI}}_{\mathbf{CT}}\left(\mathbf{PNTL}\right)}{{\mathbf{VOI}}_{\mathbf{CT}}\left(\mathbf{NTL}\right)}$
10	Lung (in case of substantial LSF)	VOI_CT_(Lung)	CT-based, to determine volume and counts.

The black VOI of Fig. 2 represents the lesion border delineated using CT; the red area represents the perfused region of the same lesion delineated on the nuclear medicine image, and the grey area represent a low-perfusion region in the tumour. Breathing motion at the liver dome shifts and deforms the liver and the lesion shapes on the nuclear medicine images, which blurs counts outside the CT-defined liver border (Fig. 2C, D). The general concept is that, in case of a mismatch, volumes should be determined on radiological images, while corresponding counts are taken from VOIs defined on the nuclear medicine images.

VOIs should be outlined by means of fused radiological and nuclear medicine images [77]. For convenience, avoid, if possible, the use of thin CT slices. A list of sequential segmentation steps is given below. Table 2 summarizes the compartments that generally need to be contoured.

In the segmentation sequence described below, subtraction of one region from another may be obtained either by Boolean operations on VOIs or by arithmetical subtraction of VOI counts or volumes. The segmentation steps should be conveniently performed in this order.

1. Window the CT image to HU between 0 and 200 and manual contour around the visible liver to determine the total liver volume (VOI_CT_(whole liver)). Regions of interest (ROIs) can be drawn on interleaved CT slices and then interpolated if the software allows. Cystic and necrotic regions due to previous treatments should be excluded from the outline by increasing the lower HU threshold to approximately 40 HU.
2. Delineate targeted lesions on CT including any low-perfusion region. It is useful to name the VOIs sequentially, such as VOI_CT_(#1), VOI_CT_(#2), etc.
3. Delineate non-targeted lesions using CT (including any low-perfusion region) as a single VOI_CT_ (non-targeted lesions) and record the total volume. This has to be subtracted from VOI_CT(whole liver)_ to obtain the total non-tumoural whole liver volume.
4. To obtain liver counts, delineate the perfused liver (PL) VOI_SPECT_(PL) using ^99m^Tc-MAA SPECT. Include counts that originate within the liver but are located outside VOI_CT_(whole liver) due to breathing motion or resolution blurring (Fig. 2C and D). Exclude gastric counts due to detached free ^99m^Tc, as well as scattered events not belonging to the liver. This step inevitably includes some degree of operator dependence and uncertainty. This uncertainty directly propagates into the calculated absorbed dose. If there is a high tumour/non-tumour ratio, very low thresholds may be necessary (1–2%).
5. Obtain the perfused liver volume VOI_CT_(PL) as the intersection of VOI_SPECT_(PL) and VOI_CT_(whole liver) (Automated Boolean intersection or manual drawing).
6. To delineate the perfused lesion portions, various situations may be encountered, as illustrated in Fig. 2. If a threshold method is used, the level can be adjusted such that the outer border of VOI_SPECT_ matches the outer border of VOI_CT_, while still excluding the low-perfusion region [77]. Absorbed dose should be reported for both VOI_SPECT_ and VOI_CT_ volumes. Where several small widespread perfused lesions are observed, these can be treated as a single volume, VOI_SPECT_(lesions).
7. Obtain counts for Perfused Non-Tumoural Liver (PNTL) in VOI_SPECT_(PNTL), starting from VOI_SPECT_(PL) obtained in step 4 and subtracting all VOIs (Boolean subtraction) or counts (manual subtraction) of VOI_CT_(targeted lesions) and VOIs or counts in CT-based VOIs for necrosis from previous treatment.
8. Determine the CT volume associated with the PNTL limiting VOI_SPECT_(PNTL) within VOI_CT_(whole liver) (Automated Boolean intersection of VOIs or manual drawing).
9. Obtain the non-tumoural whole liver volume from VOI_CT_(whole liver), subtracting (a) all VOI_CT_(target lesions); (b) the CT-based VOIs for non-target lesions and (c) CT-based VOIs for necrosis from previous treatment. The non-tumoural whole liver counts are identical to the PNTL counts. Record the non-tumoural perfused liver volume fraction Vf as the volume ratio between PNTL and the non-tumoural whole liver volume.
10. In cases of substantial lung shunt, segmentation of the lung could be conveniently accomplished on CT using a region-growing algorithm. A Hounsfield Unit upper-level threshold of − 150 was used by Allred et al. [80], while Kao et al. adjusted such threshold between − 600 HU and − 150 HU [81]. Lopez et al. propose to use the diagnostic CT for cases where the SPECT/CT AFOV do not cover the whole lung (truncated lung SPECT) [82]. If the free-breath CT coupled to SPECT is used, the individual lung mass may be obtained multiplying the volume times a nominal density of 0.3 g/cm^3^ [81], which represents an approximated value averaged over the breathing period. If the diagnostic CT is used, a more accurate value can be easily measured with more sophisticated methods [82, 83]. In ^99m^Tc-MAA SPECT/CT, lung counts are strongly overestimated because of scattered counts from the liver which contribute to lung VOI counts [82]. Ordinary scatter correction methods correct for this effect only partially. For this reason, the lung count density should be evaluated in a VOI covering the whole left lung only [84]. Then total lung counts have to be deduced by proportionality of the considered left lung volume to the total lung volume. We discourage the use field of view covering only the lower portion of the lung (truncated lung SPECT). This was proposed in only one paper, but data themselves showed a scatter influenced, apparently decreasing concentration from lung base to apex [82]. In a truncated SPECT, extrapolation of mean left lung activity concentration from its base, which is the portion most influenced by uncorrected scatter counts from liver, introduces an overestimation. For patients with substantial LSF, a field of view covering the whole lungs is therefore recommended both in pre- and post-therapy imaging.
11. Infiltrative lesions present problems, as detailed in the Image segementation of infiltrative lesions section.
12. If possible, neoplastic portal vein thrombus should be identified and dosimetrically evaluated as a lesion. Its volume should not be subtracted from the liver volume unless it is located within the organ.

*Important note:* In frequent situations like Fig. 2A, the segmentation sequence is simplified, and you can draw a single VOI for each object. For example, some of the regions defined in Table 2 can be outlined by the same VOI. The Numerical example section in the Appendix reports a numerical example of a dose calculation using the above procedure.

## Image quantification

Quantification is the method to convert counts in a nuclear medicine image to an activity distribution. To do so, a numerical factor representing the sensitivity of the system is required. There are two possible ways of determining such a factor.

### Absolute calibration method

A source of known activity is imaged in a predefined geometry (it may be a point source in air, a uniform cylindrical phantom or a complex anthropomorphic phantom). The calibration factor is deduced as the ratio between the known activity and count rate in the reconstructed image. This factor is then applied to all patient datasets. (This method is ordinarily applied to any PET scanner for ^18^F).

### Patient–relative conversion method

For this method, the known net activity injected in the patient is used for calibration. Provided all areas of uptake are visible within the AFOV, a conversion of injected activity to total image counts is possible.

This method cannot be applied to SPECT or PET quantification after systemic administrations but is applicable and quite convenient in locoregional liver administrations. The advantage of this approach is that there is no mismatch between the calibration source geometry and patient geometry, which may affect the accuracy of quantification. The patient–relative calibration method is therefore adopted by most authors for radioembolization, both in the pre-therapy and in the post-therapy imaging.

The conversion factor, CF **(**in units of GBq/counts), is given by the ratio between the total intended ^90^Y activity (pretreatment dosimetry) or the net administered ^90^Y activity (posttreatment dosimetry) and total counts (liver + lung) collected in the ^99m^Tc SPECT, ^90^Y PET or ^90^Y bremsstrahlung SPECT images. A specific conversion factor is generated for each patient, for each potential ^90^Y administration and for each tomographic acquisition (pre- and post-therapy).

### Pros and cons of the patient–relative conversion method

The patient–relative conversion method is generally the favoured of the two methods in radioembolization, both in the simulation and in the verification session. Generally, this will yield a better quantification accuracy, provided that the net ^90^Y injected activity is accurately known. In some cases, the administered activity is difficult to ascertain, for example, in patient with stasis [66]. It is advised that the patient–relative conversion factor is defined in the same way across both imaging sessions as this allows for the same formalism to be applied.

Accurate absolute image quantification through an absolute system calibration is not trivial. This is true for both ^99m^Tc and ^90^Y PET since it is affected by potential inaccuracies in the scatter and random correction methods. This was observed in studies where the known total phantom activity was not recovered using absolute system calibration [36, 66, 85]. An absolute ^99m^Tc system calibration during simulation imaging requires the activity and the time of the ^99m^Tc-MAA syringe measurement to be accurately recorded. The fractional activity of ^99m^Tc in each VOI is then necessary to calculate the potential ^90^Y activity.

The absolute ^90^Y PET scanner calibration might be favourable for situations where the measured net injected activity is unreliable.

## Basic dosimetric calculation: mean absorbed dose

### Historical lung-absorbed dose and lung shunt fraction (LSF) limits

According to historical studies concerning lung toxicity [86, 87], five over eighty patients received pneumonitis following treatment with resin microspheres. Lung-absorbed doses, presumably evaluated on planar scans, ranged from 10 to 36 Gy, median 25 Gy. The onset of radiation pneumonitis ranged from 1 to 6 months after internal radiation treatment, median 3 months. Three patients died from respiratory failure. A limit of 30 Gy to lung-absorbed dose was fixed for single administration, and 50 Gy cumulative in repeated administrations.

Dosimetric limits indicated by the manufacturer of glass spheres are identical. For resin microspheres, the LSF limit is given as a fraction of injected activity: treatment contraindication for LSF > 0.20, suggested activity reduction of 20% or 40% for 0.10 < LSF < 0.15 or 0.15 < LSF < 0.20 respectively. Note that this is based on a maximum vial activity of 3 GBq and a standard lung mass of 1 kg, which corresponds to a maximum potential lung-absorbed dose of 30 Gy.

#### Problem 1: Imaging methodology (planar vs SPECT/CT scan)

Recent literature raises concerns regarding the quantitative accuracy of planar imaging used for LSF determination [45]. In particular, the lack of attenuation and scatter corrections could result in a large overestimation of LSF_planar_, compared with more accurate LSF_TOMO_, obtained with fully corrected SPECT/CT or ^90^Y PET.

However, lung-absorbed dose limits were determined based on data acquired from planar imaging [86, 87] for a standard lung mass of 1 kg and without a Normal Tissue Complication Probability (NTCP) analysis. Using fully corrected SPECT/CT evaluations [45], the 30 Gy dose limit may not be appropriate if a systematic difference is evident between LSF_TOMO_ and LSF_planar_.

From a legislative perspective, to respect the 30 Gy limit indicated by manufacturers, the therapy team should adopt a consistent methodology (i.e. a planar scan without corrections).

The use of a more accurate additional lung tomographic scan is advised in patients with substantial lung shunt as a parallel calculation in order to prospectively collect accurate LSF_TOMO_ and absorbed dose–toxicity data. A reliable lung-absorbed dose limit in radioembolization according to this state-of-the-art methodology is not yet available. Limits may also potentially differ between resin and glass microspheres.

#### Problem 2: Weak predictive power of MAA on microsphere LSF

Recent works remark the large MAA overestimation of LSF_TOMO_ with respect to real therapeutic microspheres (^166^Ho [36] and ^90^Y glass microspheres [35]), even with accurate quantification ^99m^Tc SPECT/CT. This is attributable to the smaller size and consequently higher penetrability of the smallest MAA through capillaries compared to microspheres.

#### Problem 3: Sporadic and low-grade lung toxicity after glass microspheres

With glass spheres, only one case of pneumonitis has been reported in literature following 56 Gy. However, the patient had previous chronic lung impairment and pulmonary embolism [88]. Conversely there is evidence of mild and infrequent lung toxicity, for administrations above 30 Gy. Salem et al. [89] studied 58 patients who received a lung-absorbed dose > 30 Gy in a single treatment and 50 Gy cumulatively without developing radiation pneumonitis. Only 10/53 patients exhibited grade 1 lung toxicity. Predicted absorbed dose to lungs higher than 100 Gy were tolerated without any adverse effect.

#### Difficult to predict pneumonitis with MAA and impact on treatment

The above-mentioned problems render it difficult to accurately predict lung impairment based on an MAA scan, especially in cases with substantial lung shunt to be treated with glass spheres.

Two important therapeutic drawbacks are derived from this situation:

1. Patients could be undertreated in order to reduce the overestimated lung-absorbed dose
2. The actual activity in the liver is higher than that predicted, by the same amount that the lung activity is overestimated.

### The proposed procedure: three classes of lung shunt

**Fig. 3:**
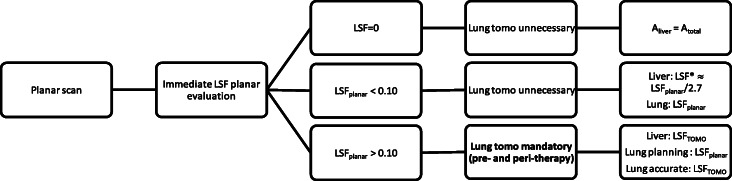
Proposed workflow of the dosimetric calculation for liver and lung to reduce the frequency of lung tomographic scans

The inclusion of the lungs may require a second SPECT/CT scan over the thorax in tall patients. This is demanding in clinical routine, when lung shunt can be considered non-clinically relevant, and the acquisition over the lung FOV can potentially be excluded. On the contrary, additional quantitative tomographic scans (pre- and peri-therapy) covering the lungs should be acquired on selected cases of substantial LSF, to prospectively record reliable quantitative data (internal consensus). The proposed workflow for the three types of lung shunt is schematically indicated in Fig. 3.

#### Patient without lung shunt (LSF = 0)

For patients without evidence of lung shunt on the ^99m^Tc-MAA image, the total intended or administered ^90^Y activity, A_total_ in the liver is

1 $$ {\mathrm{A}}_{\mathrm{total}}={\mathrm{A}}_{\mathrm{liver}} $$

Note that only ^90^Y activity enters the calculation, ^99m^Tc-MAA activity is not required.

2 $$ {\mathrm{N}}_{\mathrm{total}}={\mathrm{N}}_{\mathrm{liver}} $$

where N_liver_ are counts within the perfused liver which may be derived from ^99m^Tc SPECT, ^90^Y PET or ^90^Y SPECT. The conversion factor CF, in unit GBq/counts, is

3 $$ \mathrm{CF}=\frac{{\mathrm{A}}_{\mathrm{total}}}{{\mathrm{N}}_{\mathrm{total}}} $$

#### For all patients with lung shunt: calculation of LSF_planar_

The calculation of LSF_planar_ presented in this section should be performed for all patients showing a lung shunt on planar imaging. The methodology should be based on the historical works used to derive the dose limits. This includes planar imaging of a single anterior view, without attenuation, scatter or background correction. However, we propose at least a planar conjugate view approach with background correction. ROIs should be drawn encompassing the liver and lungs with associated background ROIs. The net number of counts N_organ_ is derived from the raw organ counts N_organ,raw_ after correction for background N_bckg_ (normalized for differences in sizes of the ROIs). A correction factor for background should also be applied according to Buijs et al [90] which is assumed equal to 0.5 for large organs (see the numerical example in the Numerical example section in the Appendix)

4 $$ {\mathrm{N}}_{\mathrm{organ}}={\mathrm{N}}_{\mathrm{organ},\kern0.75em \mathrm{raw}}-\frac{{\mathrm{N}}_{\mathrm{bckg}}}{{\mathrm{Area}}_{\mathrm{bckg}}}\times {\mathrm{Area}}_{\mathrm{organ}}\times 0.5 $$

If a dual-head gamma camera is used with the conjugate view technique, equation (4) has to be applied to the anterior and posterior images in order to obtain the net counts: N_organ ANT_ and N_organ POST_. The geometric mean of net counts in each organ is then obtained using:

5 $$ {\mathrm{N}}_{\mathrm{organ}}=\sqrt{{\mathrm{N}}_{\mathrm{organ}\ \mathrm{ANT}}\times {\mathrm{N}}_{\mathrm{organ}\ \mathrm{POST}}} $$

LSF_planar_ is then determined using

6 $$ {\mathrm{LSF}}_{\mathrm{planar}}=\frac{{\mathrm{N}}_{\mathrm{lung}}}{{\mathrm{N}}_{\mathrm{lung}}+{\mathrm{N}}_{\mathrm{liver}}} $$

Lung-absorbed dose in Gy should be calculated according to the manufacturers’ indications, using a standard lung mass of 1 kg, and activity in GBq:

7 $$ {\mathrm{D}}_{\mathrm{lung}}=49.75\times \frac{{\mathrm{A}}_{\mathrm{total}}}{1}\times {\mathrm{LSF}}_{\mathrm{planar}} $$

The value of 49.75 is detailed after equation 20.

#### Patients with non-clinically relevant lung shunt fraction (LSF_planar_ < 0.10)

Due to the poor quantitative accuracy of LSF_planar_ it is not easy to define a clinically relevant threshold, as the measure does not consider an individualized lung mass. For the most critical patients with small lung mass (0.5 kg) the historical lung-absorbed dose limit of 30 Gy is respected up to 3 GBq administered. In this case, the risk of pneumonitis should be low, and the approximation of equation (7) is sufficient for lung.

However, an unacceptable underestimation of A_liver_ from the overestimation of LSF_planar_ will expose liver to an unexpected overtreatment. See equation (8).

8 $$ {\mathrm{A}}_{\mathrm{liver}}={\mathrm{A}}_{\mathrm{total}}\left(1-{\mathrm{LSF}}_{\mathrm{planar}}\right) $$

Therefore, for evaluating liver absorbed doses it is recommended to adopt an approximated LSF* that can be obtained by dividing LSF_planar_ by a factor which varies according to the centre and to the calculation method used. Yu et al. [84] determined 3.80 ± 4.0 for all studied patients, and 2.7 ± 1.07 for patients with lung dose > 15 Gy predicted with LSF_planar_. Dittman et al. [44] reported values of 2, using linear interpolation, or 3.6 as the ratio of mean values. Lopez et al. [82] found 2.3, using linear interpolation, or 2.7 as mean relative difference. The average of these factors is 2.7.

9 $$ {\mathrm{LSF}}^{\ast }={\mathrm{LSF}}_{\mathrm{planar}}/2.7 $$

This allows to determine the liver activity more accurately than by equation (8):

10 $$ {{\mathrm{A}}^{\ast}}_{\mathrm{liver}}={\mathrm{A}}_{\mathrm{total}}\times \left(\ 1-{\mathrm{LSF}}^{\ast}\right) $$

This approximation is crude, since the individual variability around 2.7 is large. It may be accepted to reduce the liver overtreatment risk in this class of patients since it is applied where a low LSF_planar_ has been calculated. The unique possible alternative is determining LSF_TOMO_ from SPECT/CT. This is of course more accurate, but more demanding.

LSF* allows the conversion factor CF to be determined more accurately than that derived from planar imaging, even in the absence of a tomographic scan, by reverting equations (6) and (3):

11 $$ {\mathrm{N}}_{\mathrm{lungs}}^{\ast }={\mathrm{N}}_{\mathrm{liver}}\times {\mathrm{LSF}}^{\ast }/\left(1-{\mathrm{LSF}}^{\ast}\right) $$

12 $$ {{\mathrm{N}}^{\ast}}_{\mathrm{total}}={\mathrm{N}}_{\mathrm{liver}}+{{\mathrm{N}}^{\ast}}_{\mathrm{lungs}} $$

13 $$ {\mathrm{CF}}^{\ast }=\frac{{\mathrm{A}}_{\mathrm{total}}}{{{\mathrm{N}}^{\ast}}_{\mathrm{total}}} $$

The “*” sign indicates the use of approximated values, since lung counts N^*^_lungs_ are not evaluated by any tomographic imaging. Note that for cases with a non-clinically relevant lung shunt, from equation (9) we have that LSF* < 0.10/2.7, i.e. LSF* < 0.037.

#### Cases of substantial lung shunt (LSF_planar_ > 0.10)

For rare patients demonstrating a substantial LSF_planar_ > 0.10 [44], the tomographic scan should cover the whole lung volume both in the simulation and in the verification session. Accurate measurements of LSF_TOMO_ should be evaluated on fully corrected tomographic images with N_organ_ deduced from 3D VOIs over the liver and lungs, as detailed in the Segmentation sequence section and equation (6). Individual lung mass in kilograms determined on CT should be used for dosimetry. Lung counts should be deduced from a VOI on the left lung as described above.

14 $$ {\mathrm{A}}_{\mathrm{liver}}={\mathrm{A}}_{\mathrm{total}}\times \left(\ 1-{\mathrm{LSF}}_{\mathrm{TOMO}}\right) $$

15 $$ {\mathrm{A}}_{\mathrm{lungs}}={\mathrm{A}}_{\mathrm{total}}\times {\mathrm{LSF}}_{\mathrm{TOMO}} $$

16 $$ {\mathrm{D}}_{\mathrm{lung}}=49.75\times \frac{{\mathrm{A}}_{\mathrm{total}}}{{\mathrm{M}}_{\mathrm{lung}\ \mathrm{individual}}}\times {\mathrm{LSF}}_{\mathrm{TOMO}} $$

The calculation proceeds with accurate values:

17 $$ {\mathrm{N}}_{\mathrm{total}}={\mathrm{N}}_{\mathrm{liver}}+{\mathrm{N}}_{\mathrm{lungs}} $$

18 $$ \mathrm{CF}=\frac{{\mathrm{A}}_{\mathrm{total}}}{{\mathrm{N}}_{\mathrm{total}}} $$

Cases of patients with substantial lung shunt who cannot tolerate long scans to cover both liver and lungs can be calculated according to the approximation of equation (9), but activity should be chosen with maximal prudence.

### Intra-liver mean absorbed dose in macroscopic VOIs (subtraction method)

The permanent trapping of ^90^Y microspheres, discussed in the Foreword section, and the local energy deposition hypotheses (Simple voxel dosimetry with ordinary camera software section) justify the assumption that the mean absorbed dose is directly proportional to the image counts within the VOIs.

The total organ and lesion masses M_liver_, M_tumour 1,_ M_tumour 2,…,_ should be determined from volumes obtained with segmentation, correcting for the liver density of 1.05 g/cm^3^ reported in ICRP 89 [23], which may lower by 2–3% for more fatty livers, ICRU 44 [91]. The density could be made patient specific with a CT scanner-specific calibration for the conversion of mean HU to liver density. The total counts N_total_ and lesion counts N_tumour 1_, N_tumour 2,_ …, are obtained after segmentation.

^90^Y activity in a VOI is then determined using

19 $$ {\mathrm{A}}_{\mathrm{VOI}}=\mathrm{CF}\times {\mathrm{N}}_{\mathrm{VOI}} $$

The mean absorbed doses to the region is then

20 $$ {\mathrm{D}}_{\mathrm{VOI}}=49.75\times {\mathrm{A}}_{\mathrm{VOI}}/{\mathrm{M}}_{\mathrm{VOI}} $$

Where the constant 49.75 Gy kg/GBq is determined assuming a ^90^Y dose factor of 1.495 × 10^-13^ Gy kg/ (Bq·s) multiplied by the physical half life of ^90^Y, 64.053 h, and divided by ln(2).

In the package inserts for resin and glass spheres, similar constants are given as 49.67 and 50 Gy kg/GBq respectively. This slight difference depends on the assumed mean liver density and on the level of approximation adopted. This constant assumes an absorbed fraction of 1 for all volumes, which is not strictly true at smaller volumes (see section about Absorbed fraction).

Once mass and count data are extracted from the segmentation, dosimetry is easily performed:

21 $$ {\mathrm{D}}_{\mathrm{tumour}\ \mathrm{j}}=49.75\times \mathrm{CF}\times {\mathrm{N}}_{\mathrm{tumour}\ \mathrm{j}}/{\mathrm{M}}_{\mathrm{tumour}\ \mathrm{j}} $$

22 $$ {\mathrm{D}}_{\mathrm{non}-\mathrm{tumoural}\ \mathrm{liver}}=49.75\times \frac{\mathrm{CF}\times {\mathrm{N}}_{\mathrm{non}-\mathrm{tumoural}\ \mathrm{liver}}}{{\mathrm{M}}_{\mathrm{non}-\mathrm{tumoural}\ \mathrm{liver}}} $$

The name “subtraction method” derives from the fact that counts and mass of non-tumoural liver in equation (22) are obtained by subtraction of the values of all lesions from the whole liver.

Chiesa et al. [48] verified that, with a single lesion and fixed VOIs, the subtraction method is numerically equivalent to the partition model [40], as a consequence of the proportionality between VOI absorbed dose and VOI counts (equations (19) and (20)). The subtraction method has the advantage of allowing the distinct evaluation on more than one lesion, which is not possible with the partition model.

### Treatment planning using mainly non-tumoural whole liver dosimetry

Treatment planning for ^90^Y TARE based on ^99m^Tc-MAA dosimetric evaluation should aim at the optimal balance between efficacy and toxicity. However, prognosis of tumour response is affected by two major limitations. First, ^99m^Tc-MAA predictions of lesion-absorbed dose may substantially differ from the actual absorbed dose verified with ^90^Y PET, as discussed in the Prediction of ^90^Y microsphere distribution using ^99^mTc-MAA section. Second, even the relationship between post-therapy ^90^Y PET and response has a poor prognostic value, since the absorbed dose intervals of responding and non-responding lesions are largely overlapped [92, 93]. Optimization is therefore difficult, seen the weakness of one of the two factors of the balance. In similar situations, the life-threatening character of liver disease pushes to apply maximization, aiming at the maximum tolerable dose of the non-tumoural tissue [94]. This may be pursued either considering the absorbed dose to the injected non-tumoural portion, or to the whole non-tumoural volume. The knowledge developed in external beam radiotherapy (EBRT) solves this dilemma. Dawson et al. indicated that the liver exhibits a pronounced volume effect (the smaller the irradiated volume, the higher the tolerance) [95]. In other words, the liver reacts, to a good approximation, as a parallel organ. Organ failure depends on the number of inactivated subunits working independently. Dawson et al. interpolated toxicity data with the Lyman model [96] and obtained a value of n = 0.97, close to 1 (value of the completely parallel organ). For these kinds of organs, Dawson et al. stated that the simplest parameter predictive of toxicity is the mean absorbed dose, averaged over the whole functional organ volume. This concept is remarked also within the Quantec paper on liver radiation toxicity after EBRT: it indicated that mean liver-absorbed doses, and not absorbed dose–volume constraints, should be used as limits to reduce the risk of liver toxicity [97]. Dawson et al. reported also that the tolerance of liver affected by primary hepato-biliary disease is lower than metastatic liver, and that different concomitant chemotherapy regimen in the latter group may result in different tolerance. Applying additional “damage-injury” models they also stated that irradiation of less than 40% of the volume allows arbitrarily high dosage.

All these EBRT principles should be applied to ^90^Y TARE, though the absorbed dose limit cannot be transposed directly, for the marked non-homogeneity of dose deposition at microscopic level with microspheres. The non-tumoural whole liver defined on CT should be used for this calculation, including injected and non-injected lobes, and excluding target and non-targeted lesions, and necrotic regions or cysts. Using this approach, the smaller the volume fraction, Vf, of the targeted region (lobe or segment), the higher the tolerable liver absorbed dose [98]. The Lyman approach with n = 1 was proposed [48] and adopted [6] for lobar injections. It is also recommended in the same scenario by an international multidisciplinary working group about ^90^Y glass microspheres [99]. A similar approach was recently modified in the indication for ^166^Ho microspheres [100]: previous indication of absorbed dose to the treated region now refers to the whole organ. Strigari et al. adopted a similar approach (mean dose to the whole organ) to determine the NTCP curve for ^90^Y resin spheres in HCC [101].

The proposed approach is not intended for situations where Vf < 0.40 where it is too conservative. In the Appendix of reference [48], it is shown that the experimental NTCP data by Chiesa et al. (non-tumoural whole liver dose, Lyman model with n = 1) are compatible with the more refined microscopic model by Walrand et al. [102], as long as lobar injections are considered. The validity of the Lyman model ceases for volumes smaller than 40% (segmentectomy). A fraction of the liver with good function may be treated with arbitrarily high absorbed dose provided that Vf < 40% [6, 95].

### Absorbed fraction

**Fig. 4:**
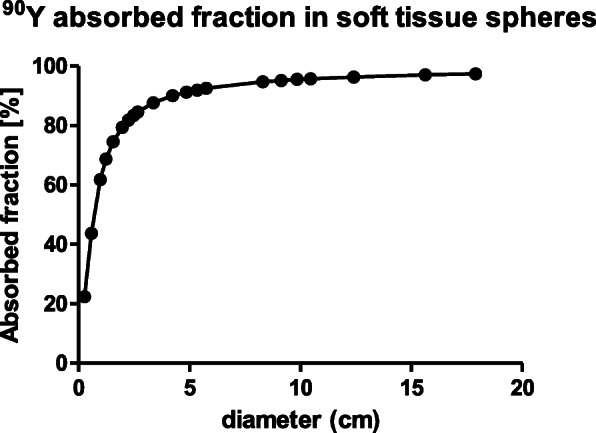
Absorbed fraction for spherical object uniformly loaded with ^90^Y (data obtained with IDAC software)

For a simulated sphere of soft tissue with density = 1.05 g/cm^3^, uniformly loaded with ^90^Y the absorbed fraction decreases with decreasing volume as shown in Fig. 4. This is not considered in the basic dosimetry of equation (20), whereby an absorbed fraction of 1 is assumed. This assumption will clearly result in an overestimate of the absorbed dose depending on volume. In general, dosimetry of lesions less than 2 cm in diameter have limited quantitative accuracy, but, as is observed here, dose to larger lesions are still potentially overestimated using this assumption.

## Voxel dosimetry

Voxel dosimetry aims to evaluate the spatial distribution of absorbed dose in a 3D image set. For each macroscopic VOI, the 3D absorbed dose map can be reduced into a 2D plot representing the distribution of absorbed dose values, in the form of differential dose–volume histograms (dDVH). The choice of the width of dDVH bins deserves some attention. Too narrow bins could be scarcely populated, and the dDVH would appear very irregular. Cumulative DVH (cDVH), intrinsically more regular, gives prompt dose volume information.

### Voxel dosimetry versus mean absorbed dose

Voxel dosimetry is recommended to complement the mean dose approach, since its superiority in nuclear medicine is still under debate [103].

Two important caveats have to be considered about the interpretation of voxel absorbed doses in nuclear medicine compared with that of EBRT.

Voxel absorbed doses generated from a nuclear medicine image will inevitably have larger uncertainty than in EBRT. This is a consequence of the image noise and partial volume effects. The level of noise depends on the counting statistics, and on the reconstruction protocol [48]. The presence of noise in a dose distribution will smoothen the shoulder and prolong the tail of the cDVH. In particular, noise severely limits the accuracy of ^90^Y PET voxel dosimetry, especially for low count regions (i.e. non-tumoural tissue).

Secondly, there is scarce data from nuclear medicine treatments, demonstrating complete response. Partial response is generally included within the response criteria. Small, isolated, under-dosed regions have limited impact on partial response and mean absorbed dose has a good predictive power [48]. Conversely, if complete response criteria were chosen as an end point, voxel dosimetry may be more suitable, allowing the identification of undertreated volumes.

### - Local deposition method (LDM) versus convolution

The LDM is a voxel dosimetry calculation method assuming no energy transport among voxels. Activity within each voxel is supposed to irradiate only the voxel in which it resides. Therefore, instead of the conventional convolution approach, described in MIRD pamphlet 17 [104], the following much simpler multiplication can be conveniently applied [48, 50]:

23 $$ {\mathrm{D}}_{\mathrm{voxel}}=\overset{\sim }{\mathrm{A}}\times \mathrm{S}\left({\mathrm{voxel}}_{\mathrm{J}}\leftarrow {\mathrm{voxel}}_{\mathrm{J}}\right) $$

**Fig. 5:**
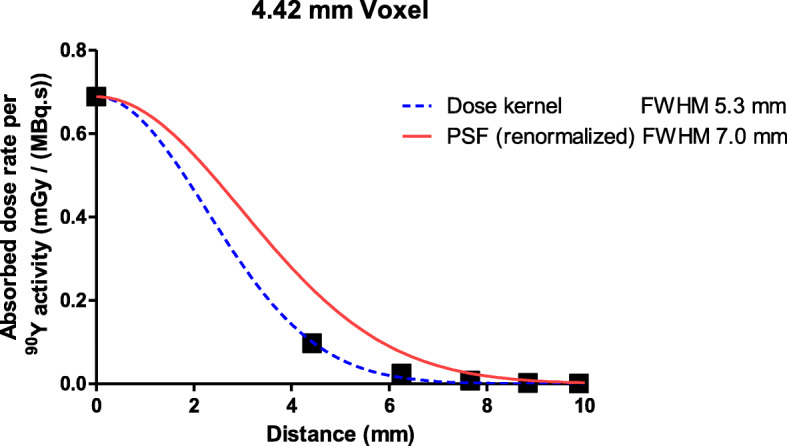
The rationale behind the local deposition method. The point spread function (red curve) was obtained with ^99m^Tc capillary source in water, OSEM (8 iterations, 8 subsets), no additional filtering [48]. Its maximum was normalized to coincide with the maximum absorbed dose kernel value. ^90^Y absorbed dose kernel values (blue dashed curve) for cubic 4.42 mm voxels from Lanconelli et al. [106].

In a simulation study using PET, Pasciak et al. [105] demonstrated that if the spatial resolution of the imaging system (FWHM of the point spread function (PSF), FWHM_PSF_) is larger than the FWHM of the S-value dose kernel (FWHM_dose kernel_), the LDM provides cDVH closer to the true cDVH. The local deposition method is therefore preferable if (Fig. 5):

24 $$ {\mathrm{FWHM}}_{\mathrm{PSF}}\ge {\mathrm{FWHM}}_{\mathrm{dose}\ \mathrm{kernel}} $$

Success of the LDM is based on the fact that in any imaging system, the limited spatial resolution misplaces emission locations from the source voxel to neighbour voxels. This misplacement serendipitously simulates the beta energy transport (Fig. 5). For ^90^Y, this phenomenon (24) is valid for clinical SPECT scanners [107, 108] and non-digital PET scanners. The exception is in the lung, where the ^90^Y beta range is larger than that in soft tissue. For digital PET scanners where the FWHM_PSF_ is in the order of 4 mm, this (24) also does not hold, as FWHM_dose kernel_ = 5.3 mm, but LDM could potentially be applied as a reasonable and convenient approximation, given the similarity of the two values. More in depth studies are required for new digital PET scanners.

**Fig. 6:**
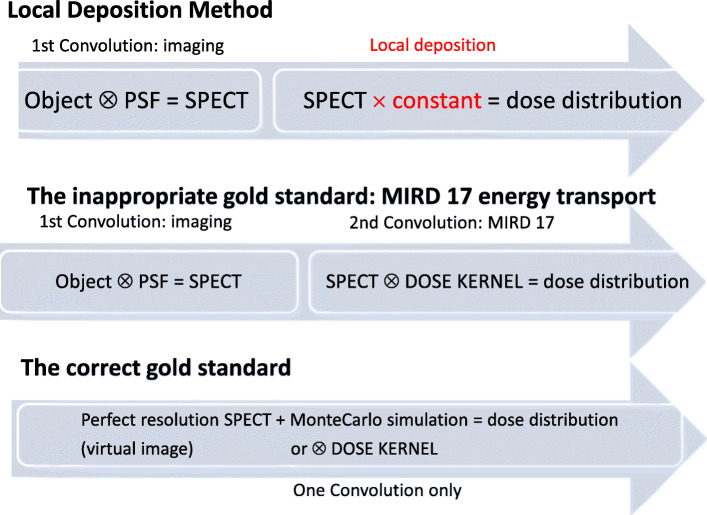
Conceptual comparison of the two possible voxel dosimetry methods with the correct gold standard

The validity of LDM is sometime tested versus a wrong gold standard. Convolution is conceptually valid if the starting image has a perfect spatial resolution (FWHM_PSF_ = 0 in a virtual image, Fig. 6). Energy transport should then ideally be simulated with the Monte Carlo method (the best gold standard) or with dose kernel convolution. However, convolution has limitations arising from density scaling, and it is prone to errors at boundaries.

When applying the LDM, only S(voxel_J_ ← voxel_J_) for self-irradiation is considered, i.e. the absorbed dose D_j_ in the voxel j per one decay, is given by the mean beta energy E_β_^MEAN^ emitted by one ^90^Y decay divided by the mass of the voxel:

25 $$ \mathrm{S}\left({\mathrm{voxel}}_{\mathrm{J}}\leftarrow {\mathrm{voxel}}_{\mathrm{J}}\right)=\frac{{{\mathrm{D}}_{\mathrm{voxel}}}_{\mathrm{J}}}{1\ \mathrm{decay}}=\frac{{{\mathrm{E}}_{\upbeta}}^{\mathrm{MEAN}}}{{{\mathrm{m}}_{\mathrm{voxel}}}_{\mathrm{J}}} $$

The voxel mass assumes a density of liver tissue ρ = 1.05 g/cm ^3^ ICRP 89 [23]. For a cubic voxel dimension of 4.42 mm, S(voxel_J_ ← voxel_J_)= 1.65 Gy/(GBq s). For alternative sizes, d [mm], S(voxel_J_ ← voxel_J_) is easily obtained by;

26 $$ \mathrm{S}\left({\mathrm{voxel}}_{\mathrm{J}}\leftarrow {\mathrm{voxel}}_{\mathrm{J}}\right)=1.65\times \frac{4.42^3}{{\mathrm{d}}^3} $$

Permanent microsphere trapping allows a further simplification:

27 $$ \overset{\sim }{\mathrm{A}}=1/\ln (2)\times {\mathrm{T}}_{\mathrm{phys}}\times {\mathrm{A}}_{\mathrm{voxel}} $$

A_voxel_ is deduced as above from the hypothesis of identical biodistributions and the patient–relative calibration, i.e. with equation 19 rewritten for a voxel with N_voxel_ counts:

28 $$ {\mathrm{A}}_{\mathrm{voxel}}=\mathrm{CF}\times {\mathrm{N}}_{\mathrm{voxel}} $$

### Simple voxel dosimetry with ordinary camera software

Using the LDM, the voxel absorbed dose is directly proportional to the voxel counts. This allows the immediate conversion of a SPECT or PET image into a 3D absorbed dose map by simple image multiplication.

29 $$ {\mathrm{D}}_{\mathrm{voxel}}=\mathrm{Q}\times {\mathrm{N}}_{\mathrm{voxel}}, $$

where

30 $$ \mathrm{Q}=1.443\times 64.053\times 3600\times \mathrm{CF}\times \mathrm{S}\left({\mathrm{voxel}}_{\mathrm{J}}\leftarrow {\mathrm{voxel}}_{\mathrm{J}}\right). $$

Once images are scaled in this way, tools can provide important voxel dosimetry information. For instance, a voxel pointer reading gives the voxel absorbed dose; an isocontour drawn gives an isodose contour. Simple VOI statistics provides maximal, minimal and mean absorbed doses. DVH may also be obtained if voxel values can be exported.

A potential truncation problem can occur when adopting this method. Image counts are usually stored in computer memory as integers. All voxels with D_voxel_ = Q × N_voxel_ < 1 Gy may therefore be rounded down to 0.

To reduce the effect of this rounding error, it may be more appropriate to use a factor 100 × Q or 10 × Q in equation (30). However, when using such small units, there is an additional risk of byte overflow. A 2-byte integer has the capacity to store values up to 2^16^-1 = 65535, which would correspond to a maximum absorbed dose of 655.35 Gy if using 100 × Q.

## Dosimetric accuracy

In the simulation session, inaccurate dose calibrator calibration of ^99m^Tc has no impact on dosimetric accuracy if the patient–relative calibration method is adopted. This is a major advantage of this method: in all VOIs, absorbed dose is deduced from the ratio of counts to the total counts. On the contrary, the uncertainty regarding ^90^Y injected activity directly propagates to the dosimetric inaccuracy (see Measurement of the ^90^Y injected activity section).

With the same method, general uncertainty originates from the VOI used to measure total counts (Segmentation sequence section). If a threshold method is used, a variation in threshold value from 9 to 12% can give rise to 13% deviation in total liver counts. This uncertainty directly propagates to the conversion factor and therefore into the absorbed dose to each VOI (equations (19), (20) and (28)).

The largest inaccuracy in dosimetric prediction is the difference between MAA and microsphere biodistribution. This was widely discussed in the Prediction of ^90^Y microsphere distribution using ^99m^Tc-MAA section, as intrinsic and operator-dependent sources of inaccuracy. All authors report that for lesions, the observed difference is unacceptably large in some patients. An example by Gnesin et al. found differences in lesions ranging between − 64 and + 250%, both with resin and glass microspheres with whole non-tumoural liver dose prediction accuracies of − 42 to + 35% for resin spheres [31].

Quantitative accuracy of small objects, with diameters less than 2 cm is primarily dominated by the Partial Volume Effect of the imaging methodology. Underestimation in ^99m^Tc-MAA SPECT is of the order of 20% with this dimension (^99m^Tc Phantom scans for reconstruction optimization section in the Appendix). According to Willowson et al., the same limit should be set for reliable ^**90**^Y PET dosimetry [66].

A size-dependent overestimation by equation (20) is due to overestimating the absorbed fraction (Fig. 4). It is interesting to note that recovery coefficients (PVE) and absorbed fractions have a similar dependence on the object size. If the two curves could be demonstrated to be identical on a system, the two errors could cancel each other out, with no need of PVE correction.

The accuracy of post-therapy ^90^Y imaging was discussed in the ^90^Y PET reconstruction section for non-digital PET and in the Dosimetry based on ^90^Y bremsstrahlung SPECT section in the Appendix for bremsstrahlung SPECT imaging.

Estimates of inaccuracy derived from operator dependent segmentation as well as from breathing motion is not available. This is well known in any kind of quantification on nuclear medicine images. The influence of breathing motion is mitigated by adopting the two-VOI method, described in this document, where volumes are derived from CT, and counts from nuclear medicine images. Methods to evaluate global dosimetric uncertainty are detailed in the EANM guidelines [57].

## Dosimetry reporting

Dosimetry reporting has two aspects: documentation for clinical use and for scientific purposes.

For the first goal, in case of planning, the medical physicist expert should develop and sign the “dosimetric report” containing 3 or 4 possible activity choices and corresponding dosimetry to segmented VOIs (see the numerical example in the Numerical example section in the Appendix).

Once the therapist has chosen the therapeutic activity, a second document (“treatment plan”) should be produced, reporting only the chosen administered activity and the corresponding dosimetry. This should be agreed and signed by the therapist and included in the official patient historical records.

A third document (“treatment verification”) should be similarly produced, reporting both predicted and actual absorbed dose values measured using ^90^Y PET.

For publication purposes, details of good dosimetry reporting are given within the EANM Dosimetry Committee guidelines [109].

## Commercially available dosimetric software

The methodology proposed in this document does not require any additional commercial software other than co-registration and VOI drawing tools, which are usually available on SPECT and PET processing workstations. A simple spreadsheet is also required (provided as online example). Once volume and count information have been evaluated for each VOI, the dosimetric computations are simple multiplications and divisions, as shown above. However, manual slice-by-slice segmentation is time-consuming, and manual subtraction of VOI counts is cumbersome in the absence of Boolean operators.

Several commercial dosimetric programs are currently available including some freeware. The advantages of this dedicated software are the inclusion of additional tools such as automated rigid or deformable registration, or automated segmentation of organ and tumours. This can have a considerable time saving for absorbed dose calculations. Immediate Boolean operations between VOIs are also available. However, some products have fixed workflows limiting the freedom of the operator. Moreover, none of them include the two-VOI method, which allows to compute the dose to a region combining volume from CT segmentation and counts from NM segmentation.

## Other guidelines

These guidelines are focused on methodology and recommendations for dosimetry. More diverse guidelines covering the wider clinical aspects of the field are given by the American College of Radiology revised in 2019 and the EANM clinical guidelines [14]. Of note is the ACR advice regarding the absorbed dose delivered by the CT scan of nuclear medicine hybrid imaging: “*The CT as a part of a SPECT/CT should be of good quality (low noise). There is limited value to using a low-dose CT scan when the liver will be treated to radiation doses that will be orders of magnitude greater.*” Herein, we recommend that the quality of CT of hybrid imaging be sufficient for segmentation.

Further recommendations of note are those by the AAPM [10]. Written by a multidisciplinary panel including medical physicists, interventional radiologists and radiation oncologist, they cover a diverse overview and a variety of fields. Topics include the rational for choice of microsphere, liver anatomy, indications and contra-indications, imaging for diagnosis and follow-up, angiographic procedures, interventional radiology, metrology of ^90^Y activity, radiation safety, instrumentation quality control, manufacturer prescription criteria and delivery procedure.

The present EANM guidance document presented as a standard operative procedure, is focused solely on dosimetry methodology and intended to offer a more in-depth description of the difficulties and approaches to dosimetry that are not covered to the same extent within other similar guidelines.

Strategies for treatment planning form a strong basis to the recommendations set out in this guideline which are not covered elsewhere. Voxel dosimetry is recommended in the final section of the AAPM document, based on convolution. We strongly endorse the mean dose approach on macroscopic regions. Voxel dosimetry is suggested for complementary use, performed using the local deposition method. This approach is much simpler and arguably more correct due to the limited spatial resolution of nuclear medicine images.

A patient–relative conversion factor is proposed for ^90^Y bremsstrahlung SPECT by AAPM. We propose its use for all dosimetric imaging, including ^90^Y PET dosimetry, which was not discussed in the AAPM guideline [10].

## Need, feasibility and convenience of dosimetric optimization in radioembolization

Dosimetry-based treatment planning could be introduced for treatment of liver lesions with radioactive microspheres, as its benefits outweigh its costs. The options for dosimetry are the most favourable among all kinds of therapy, for three basic reasons.

The life expectancy in cases of liver disease is markedly shorter than in other oncological disease treated in nuclear medicine. Treatment can seldom be repeated twice and only very rarely more so. An optimized first treatment can noticeably increase life expectancy [5]; more frequently, a successful microsphere treatment can allow bridging to lifesaving transplantation or downstaging to surgical resection.

Radioembolization without dosimetric optimization exposes patients to short-term severe or life-threatening risks (i.e. treatment-related liver decompensation and death [6]). This is significantly more than in any other nuclear medicine treatment.

Dosimetry costs for radioembolization with ^90^Y microspheres are low, since the methodology offers unique simplifications with respect to systemic radiopharmaceuticals. Only one tomographic scan is required for the absorbed dose calculation, which would ordinarily already be performed for clinical review. The additional cost of dosimetry is limited to the calculation time by physicists, and to the optional purchase of commercial software. The local energy deposition method allows for direct proportionally between absorbed dose and counts in a macroscopic VOI (mean absorbed dose approach), or to calculate absorbed dose at the voxel level. The patient–relative calibration method does not require preliminary system calibration.

The main limitation to the predictive accuracy of ^99m^Tc-MAA SPECT/CT dosimetric treatment planning is the potential mismatch between dosimetric values obtained in simulation and in therapy.

Despite such limitations, excellent correlations have been observed between pre-therapy dosimetry and clinical outcome [1–5]. These results support the prompt introduction of systematic dosimetric treatment planning.

## Conclusion and key points summary

- A single hybrid scan is sufficient for dosimetry
- Two compartment liver segmentation (tumour versus non-tumoural tissue, if possible) is the minimum level of distinction for dosimetric therapy optimization. Lung is a third compartment to be evaluated if lung shunt is present.
- Discrepancy between absorbed dose prediction with ^99m^Tc-MAA SPECT/CT and post-therapy verification is large for lesions in some patient, while it is acceptable for non-tumoural liver. Basic radiobiology of parallel organs and the volume effect indicate that the mean liver absorbed dose is a good predictor of toxicity. These two considerations push to use, as leading parameter for planning, the absorbed dose averaged over the whole non-tumoural tissue, including the non-injected portion, excluding non-functional regions. Lesion absorbed dose should anyhow be evaluated and considered in planning and verification.
- Soon after ^99m^Tc-MAA injection, all patients should be scanned with a dual head planar scintigram on the trunk, followed by SPECT/CT on the upper abdomen. If substantial lung shunt is present in planar images, SPECT/CT should also cover lungs, by a proper centring, or, if necessary, by dual field of view SPECT/CT.
- ^99m^Tc SPECT/CT should be corrected for attenuation, scatter, resolution recovery. Reconstruction protocol should be optimized on a phantom with spheres to obtain the highest recovery for small objects, without using uselessly high numbers of iteration and subsets which only increase image noise.
- ^90^Y PET scan is strongly recommended after therapeutic administration, covering the same body districts as MAA SPECT/CT, for clinical and dosimetric purposes. ^90^Y bremsstrahlung SPECT/CT accurate quantification is challenging and possible only in research centres.
- The net injected activity should be determined with a measure of the residual activity in vial, tubes and catheters.
- Segmentation should be performed on co-registered radiological and nuclear medicine images. For object with mismatch between CT and nuclear medicine images, two VOIs should be drawn on the two imaging modalities, and the corresponding counts from nuclear medicine imaging and volumes from CT should be properly combined in the dosimetric calculation.
- Patient relative conversion factor CF: quantification is obtained from total net ^90^Y activity divided by total patient counts.
- Mean absorbed dose calculation is recommended first: D_VOI_ = 49.75 × CF × N_VOI_/M_VOI_
- Dosimetry of lesions with diameter < 2 cm is prone to large uncertainties both in pre- and post-therapy imaging.
- The limit of 30 Gy to lung, historically obtained with planar imaging, is not applicable if lung dose is calculated on SPECT/CT. Planar evaluation largely overestimate tomographic values. MAA largely overestimates post-therapy lung shunt evaluation even using quantitative tomographic imaging in both sessions. Planar evaluation seems too conservative for glass spheres. The lung-absorbed dose limit is a completely open problem.
- Voxel dosimetry is proposed to collect data, but its advantages are not demonstrated yet. Local deposition method is suggested. This allows to rescale voxel counts of a tomographic image into voxel absorbed dose values by a simple image multiplication by a constant.
- Caution is necessary in interpreting nuclear medicine DVH since they are distorted by noise, especially in ^90^Y PET.
- Major sources of absorbed dose uncertainties are:
  - The random discrepancy between MAA and microsphere biodistributions especially on lesions (− 64%, + 250% in one paper),
  - The determination of the conversion factor from total counts on patient (random uncertainty)
  - Partial volume effect for lesions (systematic uncertainty)
  - The neglected size-dependent absorbed fraction (systematic uncertainty)
- Commercial software are available. They speed up the process, but sometime have fixed workflow.
- A dosimetric report should be signed by medical physicist, offering to therapist several activity choices with the corresponding absorbed dose values list.

This EANM dosimetry committee document gives guidance on the dosimetry methods and procedures in liver radioembolization with ^90^Y microspheres. Absorbed doses should be determined both pre- and post-therapies as the MAA simulation might mismatch the actual therapy distribution. Methods are highlighted to distinguish tumours from non-tumoural tissue, including lungs in cases of lung shunt, essential in optimizing the therapy. Prospective dosimetry is advised to follow mean absorbed dose approach.

### Supplementary Information

Additional file 1. NUMERICAL EXAMPLE

## Data Availability

All data and materials are included in the manuscript and the Appendix.

## Appendix

### ^99m^Tc Phantom scans for reconstruction optimization

The dosimetric acquisition protocol for the two phantoms should be identical to that described in the Importance of prediction with ^99m^Tc-MAA section for patients, except for the scan duration (time for projection) which can be lengthened in the noise phantom. Phantom images should be reconstructed with the same corrections as for clinical dosimetry, using all the efforts to obtain a quantitative SPECT. For an OS-EM reconstruction protocol, images should be reconstructed with a sequence of increasing number of updates P, starting from the clinical default values (for instance P values = 4 IT × 4 SUBS = 16, 6 × 6 = 36, 6 × 8 = 48, 7 × 8 = 56, 8 × 8 = 64, 9 × 8 = 72, 9 × 10 = 90,...) [48], keeping the number of subsets less than one fourth of the number of projection (SUBS < 30 = 120/4) [50].

For SIEMENS xSPECT Ordered Subsets Conjugate Gradient reconstruction (OS-CG), follow the company guidance of one subset, and vary the number of iterations up to 96. Do not apply the option for skeletal reconstruction.

An approximately 20-cm diameter, 20-cm height cylindrical uniform phantom should be used to determine the voxel noise N(P), defined using the coefficient of variation. Fill the cylinder with approximately 700 MBq of ^99m^Tc and acquire a SPECT for at least 90 s per angular step of 3° (about 3 h) so that Poisson fluctuations are negligible (mean counts per voxel > 10,000). Draw a centrally positioned cylindrical VOI with a radius and height 75% that of the phantom.

Recovery coefficients RC are determined using a phantom with hot spheres, at a concentration ratio of 4:1 with a total activity of 500 MBq. The largest sphere should have a diameter of at least 37 mm. For phantom preparation, use the main phantom to dilute the solution. Fill with water 1/4 of the phantom and add the total sphere volume. Inject ^99m^Tc and mix carefully. Fill spheres with this solution. Dilute the background volume filling the phantom with additional non-radioactive water.

A volume of interest (VOI) for each sphere should be drawn which reproduces the physical internal sphere volume.

For each reconstruction, plot recovery coefficients RC(V) as a function of their volumes.

The optimal choice P_opt_ is the reconstruction protocol where the RC(P) curve reaches a plateau. If this choice is ambiguous among different P, a lower value of P_opt_ is recommended as this will inevitably lead to lower image noise.

Deduce the sphere volume V_80%_ for which RC(V_80%_) ≈ 0.8. Below such volumes (typically about 3 mL, i.e. 1.8 cm diameter), the partial volume effect is significant [Chiesa et al., unpublished data, acceptance test of SIEMENS Symbia Intevo T2 SPECT/CT system with Ordered Subset—Conjugated Gradient reconstruction, 256 x 256 matrix, 72 iterations, 1 subset, no filter]. Mean absorbed doses for uniformly perfused spherical lesion may be fairly accurate *without* PVE correction above this volume. However, absorbed dose evaluated of non-spherical, non-uniform lesions smaller than V_80%_ are prone to potentially large uncertainties.

An alternative approach to reconstruction optimization was recently proposed by Siman et al. [69] for ^90^Y PET, but the concept is equally applicable to ^99m^Tc SPECT. A phantom with hot spheres in warm background (concentration ratio 4:1) is reconstructed with the sequence of P values and post-reconstruction filtering with Gaussian functions of increasing FWHM. The figure of merit for optimization is the discrepancy between the obtained cDVH in each sphere and the reference virtual cDVH obtained from the known activity distribution without noise or resolution blurring.

### ^90^Y PET reconstruction protocols

For PET image reconstruction, the following protocols are recommended [66].

- GENERAL ELECTRIC scanners: 2 iterations, 24 subsets, no additional filtering, or Q.Clear^TM^ with a high beta value (up to 1500). For Discovery 690 and 710 systems with TOF and resolution recovery applied (VPFXS), Siman et al. [69] suggest 36 updates and a Gaussian filter of 5.2 mm FWHM
- PHILIPS scanners: BLOB OS 2–3 iterations, 33 subsets, no additional filtering
- SIEMENS scanners: 2 iterations, 21 subsets, no additional filtering

The use of 3 iterations on PHILIPS systems is suggested due to noise regularization operated by the BLOG algorithm. In this respect, the new GE noise regularization algorithm (Q.Clear™) may be used but the suggested value of the weight of the subtraction term (“beta value”) should be considerably higher than that used for FDG scanning (1500 instead of 350–400). For Siemens scanners, the NETTRUES modality should not be used since truncation of negative pixel values after delayed random sinogram subtraction produces an under-correction that may lead to large overestimation of the real activity [66, 70].

### Dosimetry based on ^90^Y bremsstrahlung SPECT

Dosimetry based on quantitative ^90^Y SPECT imaging is challenging since the bremsstrahlung energy spectrum is considerably different from typical SPECT-imaging radionuclides, with a low yield of bremsstrahlung photon emissions distributed continuously over a broad range of energies. Owing to the lack of a single-energy photo-peak and a modest count rate, a comparably broad energy window is commonly used. The detected counts in this energy window is then a mixture of counts caused by so-called primary photons, i.e. those that pass un-scattered from the decay position in the patient to the camera crystal, and photons that before interaction in the crystal may have scattered in the patient, in the collimator or material behind the crystal, or have penetrated the collimator septa. Elaborate methods have been developed with the aim of determining the amount and position of the primary photons, which are those that carry valid quantitative information about the activity distribution. Table 3 in the Appendix summarizes acquisition parameters and reconstruction settings for recent studies focusing on quantitative imaging with ^90^Y.

**Table 3:** Summary of imaging systems, choice of collimator, energy-window settings and reconstructions used in studies of quantitative ^90^Y bremsstrahlung SPECT

Study	Imaging system	Collimator	Energy window (keV)	Reconstruction settings
Ito et al. [110]	Picker PRISM-2000XP	Medium energy	Various. 57–232 concluded for clinical imaging	OS-EM (& FBP). Att.Corr: Chang method Scat.Corr: not included CRF not included
Minarik et al. [111, 112]	GE SPECT/CT Discovery VH	High energy	105–195	OS-EM Att.Corr: CT-based, mean mass-attenuation coefficient (abundance-weighted mean energy) Scat.Corr: Effective Scatter Source Estimation (ESSE) CRF included
Elschot et al. [113]	Siemens Symbia T16 SPECT/CT	High energy	50–250	OS-EM Att.Corr: CT-based, Monte-Carlo calculation embedded in reconstruction, including modelling of attenuation and scatter. CRF included
Rong et al. [114, 115]	Philips Precedence SPECT/CT	High energy	100–500	OS-EM Att.Corr: CT-based with energy-dependent effective attenuation coefficient Scat.Corr: ESSE CRF included
			Various. 80–180 found optimal	
Dewaraja et al. [116]	Siemens Symbia T6 SPECT/CT	High energy	105–195	OS-EM Att.Corr: CT-based with mass attenuation coefficient for 150 keV Scat.Corr: Monte Carlo calculation embedded in reconstruction CRF included

*CRF* collimator response function

Generally high energy (HE) or possibly medium energy (ME) collimators are used [117] to discriminate the high-energy photons in the bremsstrahlung spectrum. The application of an iterative reconstruction, such as the ordered subsets expectation maximization (OS-EM), is required to include the proper image corrections. Attenuation correction should in principle take into account each photon energy across the energy window, and different approaches have been used such as Monte Carlo calculation embedded in the projector [113], or dense energy binning [114, 115]. For ^90^Y microsphere activity quantification, good results have also been obtained by the use of a single attenuation map [116]. Due to the proportionally large contribution of scatter in the energy window, the scatter correction is particularly challenging and advanced methods such as the effective-source scatter estimation (ESSE) [115] or Monte Carlo simulations in the forward projector [113] have proven useful.

Dosimetry has also been performed based on ^90^Y bremsstrahlung imaging. The earlier work focused on dosimetry for ^90^Y ibritumomab tiuxetan (Zevalin®), while dosimetry evaluations for ^90^Y microspheres have later emerged. Assessment of the accuracy of this approach has been studied in both phantom and patient studies, as summarized in Table 4 in the Appendix.

**Table 4:** Summary of ^90^Y dosimetric phantom and patient studies

Study	Type of study	Results
Minarik et al. [111, 112]	Physical phantom	Activity error for liver and 113 mL sphere < 11%
	Patient study (^111^In and ^90^Y Zevalin)	Comparison to absorbed doses from quantitative ^111^In SPECT/CT (3 patients) Liver, spleen, kidneys within 30% Lungs within 66%
Elschot et al. [113]	Patient study (^90^Y microspheres). Physical phantom	Comparison to absorbed doses from ^90^Y PET/CT (5 patients): ROIs in liver within approximately 15–20%. ROIs in six-sphere phantom within approximately 5% (mean of all spheres). N.B. Data estimated from diagram
Rong et al. [114, 115]	Phantoms, physical and simulated for ^90^Y Zevalin	Physical phantom: activity error for 3 hot spheres in warm background < 10%. XCAT MC study: activity error < approximately 12% for spleen, liver, kidneys, heart and lungs
Dewaraja et al. [116]	Physical phantom	Activity error for 3 hot spheres (or similar) in warm background < 15%, liver and lung < 4%

As noted, the reported activity errors for liver, lesions and lung are for the latter studies within approximately 10–20%, thus making liver and lesion dosimetry, as well as lung shunt follow-up feasible. ^90^Y microsphere treatments is particularly well suited for bremsstrahlung SPECT/CT imaging, owing to the local activity administration that makes the activity concentration higher than in systemically administered therapies, such as ^90^Y Zevalin. However, these results have been obtained at academic centres, with careful attention to the physics involved. Of further interest is also the extensive developmental work concerning both design of optimal collimators and bremsstrahlung imaging by pinhole SPECT during the ^90^Y microsphere administration [119].

### Numerical example

The numerical example in the form of a spreadsheet can be downloaded from additional materials. It is conceived for a glass sphere treatment. However, it can be used for a resin treatment reducing the decay interval to 1 day or less.

Authors decline any responsibility from its use.

## Abbreviations

^18^F-FDG: ^18^F Fluoro-deoxy-glucose
AC: Attenuation corrected
AFOV: Axial field of view
A_VOI_: Activity in the VOI under study
BSA: Body Surface Area method of activity choice
CBCT: Cone beam CT
CT: Computed tomography
CF: conversion factor = total intended or net administered ^90^Y activity / total fully corrected counts
CRF: Collimator response function
dDVH: Differential dose–volume histogram, i.e. the plot of distribution of absorbed doses among voxels in a VOI dose–volume histogram
cDVH: Cumulative dose–volume histogram, i.e. the integral of cDVH from the absorbed dose considered to infinite dose
d: Cubic voxel side length in millimetre
D_VOI_: Mean absorbed dose in the VOI under study
D_VOXEL_: Absorbed dose in a voxel
EBRT: External beam radiation therapy
EM: Emission
ESSE: Effective scatter source estimation (a scatter correction method)
FOV: Field of view
FWHM: Full width half maximum
HCC: Hepatocellular carcinoma
HE & ME: High-energy & medium-energy collimators
HU: Hounsfield units
#IT: Number of iterations in OSEM reconstruction
LDM: Local deposition method without convolution
LEHR collimators: Low-energy high-resolution collimators
LEUHR collimators: Low-energy ultra-high resolution collimators
LSF: Lung shunt fraction
LSF*: Lung shunt fraction obtained after division by an average attenuation correction factor of 2.7
LSF_planar_: Lung shunt fraction obtained from planar imaging without attenuation nor scatter correction
LSF_TOMO_: Lung shunt fraction obtained from attenuation and scatter corrected tomography
MAA: Albumin macro aggregates
MIRD: Medical internal radiation dose
MIA: Maximum ^90^Y injectable activity
mRECIST: Modified RECIST
MRI: Magnetic resonance imaging
NAC: Non attenuation corrected
N_VOI_: Number of counts in the VOI under study
OS: Overall survival
OSCG: Ordered subset conjugated gradient iterative reconstruction
OSEM: Ordered subset expectation maximization iterative reconstruction
P: Number of updates, i.e. product on number of iteration times the number of subsets in OSEM reconstruction
P_opt_: The experimentally optimized P
PET: Positron emission tomography
PFS: Progression free survival
PL: Perfused liver
PNTL: Perfused non-tumoural liver
PSF: Point spread function
PVE: Partial volume effect
Q: Scale factor to convert voxel counts in absorbed dose value
RC: Recovery coefficient, dependent on the hollow sphere volume and on the number of updates
ROI: Region of interest
RR: Resolution recovery
SPECT: Single photon emission computed tomography
#SUBS: Number of subsets in iterative reconstruction
SUVmax: Maximum voxel value of standardized uptake value
TARE: Trans arterial radio embolization
TOF: Time of flight PET modality
Vf: Non-tumoural liver treated volume fraction. Vf is defined as the volume ratio between PNTL and the whole non-tumoural liver volume
VOI: Volume of interest
VOI_CT_: Volume of interest drawn on the bases of CT images
VOI_SPECT_: Volume of interest on the bases of SPECT images
WHO: World Health Organization radiological criteria
